# Chiral Twist Interface Modulation Enhances Thermoelectric Properties of Tellurium Crystal

**DOI:** 10.1002/advs.202402147

**Published:** 2024-07-23

**Authors:** Stanley Abbey, Hanhwi Jang, Brakowaa Frimpong, Van Quang Nguyen, Jong Ho Park, Su‐Dong Park, Sunglae Cho, Yeon Sik Jung, Ki‐Ha Hong, Min‐Wook Oh

**Affiliations:** ^1^ Department of Materials Science and Engineering Hanbat National University Yuseong‐gu Daejeon 34158 Republic of South Korea; ^2^ Department of Materials Science and Engineering Korea Advanced Institute of Science and Technology (KAIST) Daejeon 34141 Republic of South Korea; ^3^ Department of Physics and Energy Harvest‐Storage Research Center University of Ulsan Ulsan 44610 Republic of South Korea; ^4^ Thermoelectric Conversion Center Creative and Fundamental Research Division Korea Electro Technology Research Institute (KERI) Changwon 51543 Republic of South Korea

**Keywords:** anisotropy, chiral twist, grain boundary engineering, thermoelectric

## Abstract

Manipulating the grain boundary and chiral structure of enantiomorphic inorganic thermoelectric materials facilitates a new degree of freedom for enhancing thermoelectric energy conversion. Chiral twist mechanisms evolve by the screw dislocation phenomenon in the nanostructures; however, contributions of such chiral transport have been neglected for bulk crystals. Tellurium (Te) has a chiral trigonal crystal structure, high band degeneracy, and lattice anharmonicity for high thermoelectric performance. Here, Sb‐doped Te crystals are grown to minimize the severe grain boundary effects on carrier transport and investigate the interface of chiral Te matrix and embedded achiral Sb_2_Te_3_ precipitates, which induce unusual lattice twists. The low grain boundary scattering and conformational grain restructuring provide electrical‐favorable semicoherent interfaces. This maintains high electrical conductivity leading to a twofold increase in power factor compared to polycrystal samples. The embedded Sb_2_Te_3_ precipitates concurrently enable moderate phonon scattering leading to a remarkable decrease in lattice thermal conductivity and a high dimensionless figure of merit (zT) of 1.1 at 623 K. The crystal growth and chiral atomic reorientation unravel the emerging benefits of interface engineering as a crucial contributor to effectively enhancing carrier transport and minimizing phonon propagation in thermoelectric materials.

## Graphical abstract

Tellurium has a chiral trigonal crystal structure, high band degeneracy, and lattice anharmonicity. Sb‐doped Te crystals contain the interface of chiral tellurium (Te) matrix and embedded achiral Sb_2_Te_3_ precipitates, which induce unusual lattice twists. The chiral atomic reorientation unravels the emerging benefits of interface engineering as a crucial contributor to effectively enhancing carrier transport and minimizing phonon propagation in thermoelectric materials.

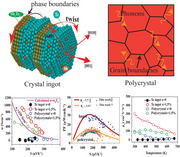

## 1 Introduction

Thermoelectric materials and devices enable the direct conversion of waste heat to electricity for sustainable environmental energy management.^[^ ^1^ ^]^ The conversion efficiency of thermoelectric material is measured by the dimensionless figure of merit *zT* = *S* ^2^σ*T*/κ_*tot*_, where *S* is the Seebeck coefficient, σ is electrical conductivity and *T* is the absolute temperature and κ_*tot*_ represents the total thermal conductivity. Here κ_*tot*_ is the sum of lattice (κ_*lat*_) and electronic (κ_*ele*_) thermal conductivities. Over the years, several strategies have been employed to enhance the power factor (*S* ^2^σ) including modulation doping,^[^ ^2^ ^]^ energy filtering of carriers,^[^ ^3^ ^]^ microstructural engineering,^[^ ^4^ ^]^ resonant levels,^[^ ^5^ ^]^ band convergence,^[^ ^6^ ^]^ and band nestification.^[^ ^7^ ^]^ The lattice thermal conductivity is suppressed by point defects,^[^ ^8^ ^]^ dislocations,^[^ ^9^ ^]^ nanostructures,^[^ ^10^ ^]^ and interfaces.^[^ ^11^ ^]^ These defect structures create lattice mismatches at the interfaces which significantly degrade carrier mobility (µ_*H*_). However, single crystals such as Mg_3_Sb_2_,^[^ ^12^ ^]^ GeTe,^[^ ^13^ ^]^ SnSe,^[^ ^14^ ^]^ and Bi_2_Te_3_^[^ ^15^ ^]^ have shown significant increases in carrier mobility compared to polycrystals. Alternatively, semicoherent precipitates in PbSe ^[^ ^16^ ^]^ and PbTe^[^ ^17^ ^]^ facilitate carrier transfer between adjacent grains reducing carrier scattering. Thus, growing crystals with low grain boundaries provide opportunities for enhancing thermoelectric properties.

Chiral Te (Space group, P3_1_21) exhibits a 3_1_ screw symmetry along the c‐axis, which is largely associated with Eshelby‐like twist‐driven screw dislocation and 1D helical growth.^[^ ^18^ ^]^ Such orientation‐dependent growth promotes anisotropic thermal conductivity, high carrier mobility, and interesting properties related to spin texture,^[^ ^19^ ^]^ and gyrotrophic effects.^[^ ^20^ ^]^ As expected, the single crystal and chiral orientation (001) of Te will exhibit superior electrical and atomic transport.^[^ ^21^ ^]^ However, single crystal Te is not suitable for thermoelectric applications since it exhibits p–n–p bipolar conductivity due to the low carrier concentration (≈10^16 ^cm^‐3^).^[^ ^22^ ^]^ Nevertheless, Sb‐doping suppresses bipolar conductivity, and increases electrical conductivity and the carrier concentration (≈10^19^ cm^−3^) for Te single crystals.^[^ ^23^ ^]^ So far, the enhancement in zT≈1 for polycrystalline Te has been achieved by optimizing the carrier concentration in the orders of ≈10^19^ cm^−3^ with dopants containing pnictogens: As, Bi, and Sb.^[^ ^7^, ^24^ ^]^ It is also noteworthy that the addition of dopants such as Se, Ag, Sn, Pb, and Ge only yields a marginal enhancement in thermoelectric properties.^[^ ^24^, ^25^ ^]^ However, the low solubility (≤ 0.1at %) of the pnictogens leads to the formation of telluride precipitates (As_2_Te_3_, Bi_2_Te_3,_ and Sb_2_Te_3_) which have a favorable semicoherent relationship with Te matrix and can be leveraged to enhance the electronic transport and minimize thermal conductivity. As of now, detailed studies that comprehensively address the complexity of transport properties remain scarce. It is imperative to correlate the impact of dopant distribution, the effect of grain boundaries, and microstructure on the resulting thermoelectric properties of Te.

High carrier mobility can be obtained in elemental Te via crystal growth and grain boundary twist to minimize the carrier scattering effect.^[^ ^26^ ^]^ Grain boundary lattice twist has been realized using chiral dopants and grain boundary phases.^[^ ^27^ ^]^ Second, secondary precipitates of similar crystal structure to the host matrix can significantly lower the lattice thermal conductivity and create semicoherent interfaces for minimizing carrier scattering.^[^ ^28^ ^]^ For example, grain boundary twisting creates dense dislocations arrays in Bi_0.5_Sb_1.5_Te_3_ for low lattice thermal conductivity and carrier filtering.^[^ ^29^ ^]^ For this to happen, the dangling Te bonds of the quintuple Sb_2_Te_3_ act as nucleation sites allowing lattice twist reorientation of Te atoms to form an electrical favorable semicoherent interface Sb_2_Te_3_/Te structure.^[^ ^29a^ ^]^ The lattice twisting is guided by several factors such as intrinsic chirality of Te atoms,^[^ ^30^ ^]^ Eshelby twist,^[^ ^31^ ^]^ axial screw dislocations,^[^ ^32^ ^]^ and dislocation strain.^[^ ^33^ ^]^ We hypothesize that via‐crystal growth we minimize grain boundary scattering which ensures high carrier mobility and power factor of 30 µWcm^−1^ *K* ^−2^ while actively maintaining low phonon propagation.

In this work, we grow Sb‐doped Te crystals to establish consistent effects of chiral twisting and low grain boundary scattering, as the origin for improved carrier mobility, enhanced electrical conductivity, and increase in power factor. In addition, a ≈25% reduction in thermal conductivity, in Te crystals compared to polycrystalline Te. We examined the interface between Te/Sb_2_Te_3_ at various compositions and addressed the induction of twist‐like grain rearrangement morphology along (00*l*). Accordingly, aberration‐corrected scanning transmission electron microscopy (cs‐STEM) in conjunction with the SAED pattern demonstrates lattice twisting from Sb_2_Te_3_‐rich precipitates and the intrinsic chiral structure of Te. This important observation emphasizes the beneficial role of modeling atomic interface and grain boundary engineering toward enhancing the transport properties of chiral inorganic materials.

## 2 Results and Discussion

### 2.1 Crystal Structure, Phase Purity, and Anisotropic Property

**Figure 1:**
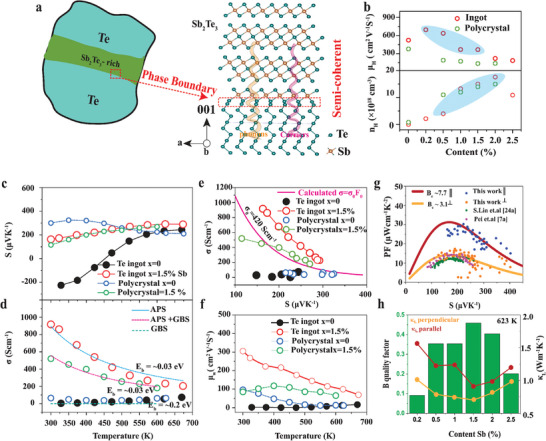
Anisotropic transport and carrier scattering mechanism. a) Semicoherent phase boundary interface between Sb_2_Te_3_‐Te matrix with simulated atomic structure of interface along the (001) b) comparing the Hall mobility and carrier concentration at various Sb content with polycrystal samples^[^ ^24a^ ^]^ c) temperature‐dependent Seebeck coefficient d) temperature‐dependent electrical conductivity with calculated acoustic phonon propagation (APS, blue dot), grain boundary scattering and acoustic phonon dependence (APS+GBS, magenta dot) and grain boundary scattering (GBS, green dot) e) Jonker plot of Seebeck and electrical conductivity with calculated intrinsic electrical conductivity of Te (magenta, line) f) weighted mobility against temperature g) power factor against Seebeck coefficient with B_e_ electronic quality factor h) anisotropic lattice thermal conductivity and material quality factor (B).

The anisotropic transport properties of Sb‐doped Te were measured as shown in Figure S1a (Supporting Information). Laue diffraction shows a highly oriented lattice along the parallel growth direction (Figure S1b, Supporting Information) but along the perpendicular direction, the lattice is polycrystalline as confirmed by the XRD pole figure density profile (Figure S1c,d, Supporting Information). Laue pattern shows slight astigmatism of the diffraction spots indicating the presence of defects due to precipitates in the single crystal Te. High thermoelectric performance can be achieved along specific crystallographic orientations with optimum carrier concentration due to low grain boundary scattering and semicoherent interfaces.^[^ ^13^, ^16^ ^]^ Tellurium (Te) has an anisotropic chiral crystal structure creating atomic layers with a high Seebeck coefficient of 400 µVK^−1[^ ^34^ ^]^ as shown in (Figure S2a, Supporting Information). By adding Sb dopants to the Te matrix, a layered Te‐Sb_2_Te_3_‐Te phase boundary is formed as illustrated in **Figure** **1**a with typical hexagonal Moiré superlattice confirming the relative twist of atomic layers along the (001) due to screw dislocations mediated growth (Figure S2b, Supporting Information). The simulated atomic structure model suggests that the orientation of the peripheral Te atoms of the Sb_2_Te_3_ structure have a semicoherent relationship with the Te matrix, forming a Te─Te homointerface along (00l). The orientation of crystals plays a critical role in determining the grain boundary density and consequently the carrier mobility. As such the highly oriented crystals have low grain boundaries which enhance the carrier mobility (x< 0.25), as illustrated in Figure 1b, and compared to polycrystalline ingots.

The Sb‐doped Te crystals exhibit degenerate p‐type characteristics with a carrier concentration of ≈10^19 ^cm^−3^ in Figure 1c. However, pure Te shows temperature‐dependent mixed conduction from n‐ to p‐type at 447 K with carrier concentration of ≈10^16 ^cm^−3^ confirmed by Hall measurement (Figure S3, Supporting Information).^[^ ^22^, ^23^ ^]^ This typical single‐crystal behavior is calculated in our previous report by density functional theory (DFT).^[^ ^26^ ^]^ By employing the two‐phase model the experimental total electrical conductivity *(σ)* is calculated as contributions from both grain and grain boundaries according to the first term and second terms, respectively in Equation 1 ^[^ ^35^ ^]^:

(1) $$\sigma_{Total}^{-1}={\left(A_{1}T^{-1.5}\right)}^{-1}+{\left[A_{2}T^{-0.5}exp\left(\frac{-E_{b}}{K_{B}T}\right)\right]}^{-1}$$

(2) $$\sigma_{o}=\frac{8\pi e{\left(2m_{e}K_{B}T\right)}^{3/2}}{3h^{3}}\mu_{w}=B_{E}/S_{o}^{2}$$

(3) $$\begin{array}{ccc} \mu_{w} & = & \frac{3h^{3}\sigma }{8\pi e{\left(2m_{e}K_{B}T\right)}^{3/2}}\left[\frac{exp\left[\frac{\left|S\right|}{K_{B/e}}-2\right]}{1+exp\left[-5\left(\frac{\left|S\right|}{K_{B/e}}-1\right)\right]}\right.\\ & & \left.+\frac{\frac{3}{\pi^{2}}\;\frac{\left|S\right|}{K_{B/e}}}{1+exp\left[5\left(\frac{\left|S\right|}{K_{B/e}}-1\right)\right]}\right] \end{array}$$

where *A_1_* and *A_2_* are the phase fraction in the grain and grain boundaries, *E_b_*, *K_B_*, *S*, *m_e_*, *e*, *h*, and *T* are a potential barrier, Boltzmann constant, Seebeck coefficient, electron mass, electron charge, Planck constant, and temperature, respectively. The electrical conductivity of the crystal ingots in Figure 1d decreases with temperature and is dominated by acoustic phonon scattering (APS). The high electrical conductivity of crystal ingots (960 Scm^−1^) compared to polycrystals (520 Scm^−1^) at 300 K is associated with low grain boundary scattering (GBS). It is evident from Equation 2 that intrinsic conductivity (σ_*o*_), weighted mobility(µ_*w*_), and electronic quality factor (*B_E_*) can elucidate the scattering phenomena. The Jonker plot in Figure 1e shows that Sb‐doped Te crystal has high electrical conductivity above the intrinsic value of σ_0_ = 420 Scm^−1^ compared to polycrystals. Here, the intrinsic electrical conductivity represents the unique conductivity σ_0_ for achieving the maximum power factor in thermoelectric materials.^[^ ^36^ ^]^ Semicoherent interfaces can be observed in both Te crystal ingots and polycrystal samples with Sb_2_Te_3_ precipitates (Figure S4, Supplementary Information).^[^ ^25^, ^28^ ^]^ Thus, high electrical conductivity in Sb‐doped crystal ingots is associated with low grain boundary scattering (GBS) carriers. As a result of low grain boundary scattering and highly oriented lattice in crystal ingots, the weighted mobility (µ_*w*_)^[^ ^37^ ^]^ calculated by Equation 3 is enhanced to ≈305 cm^2^V^−1^S^−1^ as shown in Figure 1f, compared to ≈100 cm^2^V^−1^S^−1^ in polycrystals.

The estimation of anisotropic transport properties is elucidated by the electronic quality factor *(B_E_*)^[^ ^38^ ^]^ in Figure 1g. The B_E_ measures the interdependencies of the power factor and Seebeck coefficient concerning electronic transport and predicts the influence of additional scattering mechanisms. A high *B_E_* signifies the maximum thermoelectric figure of merit, *zT*, and low carrier scattering. Interestingly, the B_E_ reaches ≈7.7 along the parallel direction for Te ingots compared to ≈3.1 in polycrystalline. As a result, we obtained a remarkable power factor of ≈30 µWcm^−1^K^−2^ along the parallel growth direction. This represents a two fold increase over the entire temperature range compared to polycrystalline ingots. Considering the anisotropic layered microstructure (Figure S2c, Supporting Information), the ingots exhibited high lattice thermal conductivity anisotropy of ≈1.5. We observed a remarkable reduction in lattice thermal conductivity along the parallel growth direction for 1.5 at. % Sb in Figure 1h. Thus, Sb optimizes carrier concentration and leads to the formation of Sb_2_Te_3_ which has a strong influence on carrier transport ($\mu_{w}/K_{L}$) as correlated by the material quality factor (B) of ≈0.45 at 623 K.

### 2.2 Morphological and Structural Characterization

**Figure 2:**
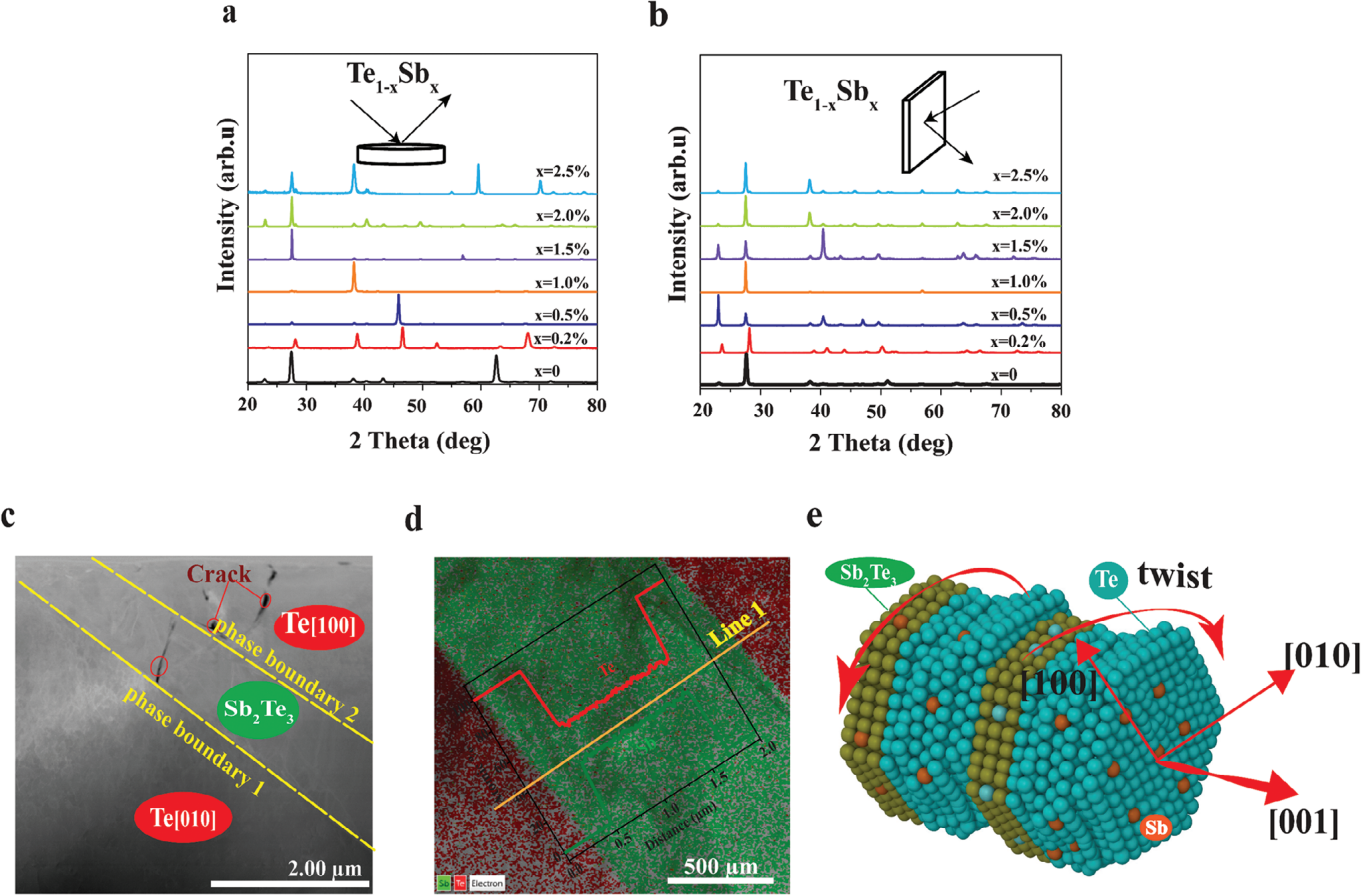
Characterization of Sb‐doped Te ingots a) XRD patterns of bulk ingot Te_1‐x_Sb_x_ in the perpendicular (┴) b) parallel (⫽) XRD diffraction c) HAADF‐STEM image for x = 0.5% Sb‐doped Te ingot showing phase boundary 1 and phase boundary 2 d) TEM‐EDS map and corresponding inset: line scan along the phase boundary e) Conceptual strategy of chiral twist and lattice rotations in 0.5% Sb‐doped Te along the [001].

**Figure** **2**a,b show the X‐ray diffraction pattern (XRD) for bulk Te_(1‐x)_Sb_x_ (x = 0‐0.25%) in the perpendicular (┴) and parallel (⫽) directions, respectively. The sharp diffraction peaks of all crystal ingots are matched to a standard PDF card (PDF# 00‐004‐0554) with trigonal structure (space group P3_1_21). The room temperature powder x‐ray diffraction (XRD) shows the presence of Sb_2_Te_3_ and Bi_2_Te_3_ precipitates (Figure S5a,b, Supporting Information) and is confirmed by SEM‐EDX (Figure S6 and Table S1, Supporting Information). The calculated lattice parameter does not show any significant differences with Sb and Bi doping (Figure S5c,d, Supporting Information). Even though the systematic increment of the Sb_2_Te_3_ precipitation with the Sb doping was not detected for the whole doping range, the increment was observed for the 1 at % and 1.5% Sb content. The relative intensities of the main XRD peaks shift to 45.9^○^ at *x* ≤ 0.5 at. % Sb content corresponding to (001) high‐oriented lattice texture. A Lotgering factor (L_f_) of 1 is attributed to single crystal orientation. The estimated degree of preferential orientation is close to unity (L_f(003)_ = ≈0.91) for x≤ 0.5% Sb content. As proof of concept, several compositions of Bi‐doped Te were prepared to confirm the preferential growth in the [001] at 0.5 at. % (Figures S7 and S8, Supporting Information). Such microstructural changes are attributed to the reorientation of Te atoms at low surface energy states along the peripheral Te atoms of preformed Sb_2_Te_3_ due to the concerted effect of capillary force and high diffusivity of Te atoms.^[^ ^29a^ ^]^

### 2.3 Chiral Twist and Grain Structure Modulation

We performed transmission electron microscopy (TEM) analysis to investigate the twisting morphology and evolution of texture in two samples (x = 0.5 at% and 1.5% at%). The high‐angle annular dark field (HAADF) image in Figure 2c shows a plate‐like morphology with sharp contrast in composition for the Te and Sb_2_Te_3_ phases. The varying composition distribution across the grains was confirmed by TEM‐EDS mapping in Figure 2d and the corresponding line scan (inset). The line profile along the phase boundary is shown in (Figure S9, Supporting Information). It is worth noting that the Sb_2_Te_3_ phase biases the seemingly amorphous Te layers into a compressive state since Sb_2_Te_3_ (32.5 GPa) has twice the shear modulus of Te (16 GPa). As such, the reorientation of Te nuclei forms a Te─Te homointerphase with the peripheral Te reactive surfaces of quintuple Sb_2_Te_3_ structure shown by the schematic of the twisted atomic structure Te [010]‐Sb_2_Te_3_ [001]‐Te [100] in Figure 2f. It must be emphasized that the formation of a sharp collinear phase boundary between the Te grains is established by twisting behavior and atomic rearrangement, and is distinct from conventional grain boundary defects in polycrystal samples which allows strong carrier scattering and suppression of phonon transport.

**Figure 3:**
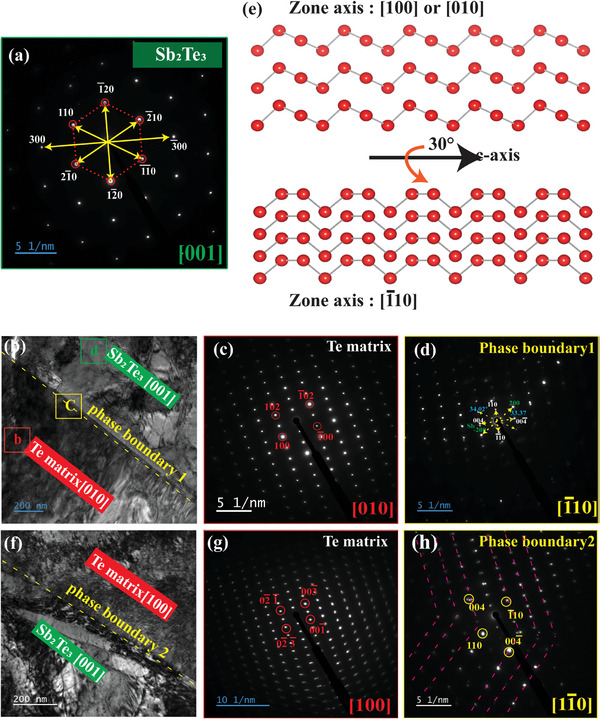
Characterization of Sb‐doped Te crystal (x = 0.5 at%) a) Hexagonal SAED pattern of Sb_2_Te_3_ in [001] zone axis b) low magnification TEM of 0.5% Sb‐doped Te ingot with phase boundary 1 c) SAED of Te matrix [010] d) SAED at the phase boundary 1e) Simulated chiral atomic models along Te‐ [$\bar{1}10$] zone axis, Te‐ [100] zone axis. f) low magnification TEM of Te phase boundary 2 g) SAED of Te matrix [100] h) SAED at the phase boundary 2.

**Figure** **3** displays the corresponding selected area electron diffraction (SAED) pattern of the Sb_2_Te_3_ precipitate which shows a perfect hexagonal structure along the zone axis of [001]. The low magnification TEM image of Te [010] in Figure 3b and STEM image (Figure S10, Supporting Information) confirms Te atoms are oriented along the [010] zone axis. However, observing the semicoherent interface phase boundary 1, between Te[010]/Sb_2_Te_3_[001] indicates that the Te atoms are twisted about the [$\bar{1}10$] axis ≈ $30^{\circ }$ clockwise of the initial orientation of Te [010] shown by SAED in Figure 3c,d. Also, the low magnification TEM for Te [100] in Figure 3f and the corresponding SAED pattern in Figure 3g show that the Te atoms are oriented along the [100] zone axis. On the contrary, the SAED along the sharp collinear phase boundary 2, Te[100]/Sb_2_Te_3_[001] in Figure 3h shows the Te atoms are oriented in [1$\bar{1}0$] zone axis which represents  ≈ $30^{\circ }$ anticlockwise rotation. In principle, from chiral atomic handedness for the trigonal system, the [100] and [010] zone axes are symmetrically comparable.^[^ ^39^ ^]^ This implies that the conformational changes create an imperfect single crystal with low grain boundaries scattering due to the pseudo‐periodic grain orientation between Te [100] and Te [010]. In Figure 3e the atomic structure model shows the chiral‐twist and atomic orientation relationship between the Te‐ [010], Sb‐ [001], and Te‐ [100] is achieved by the chiral rotation along the [$\bar{1}10$] zone axis. To confirm chiral behavior, we employed circular dichroism (CD) which measures the relevant chiroptical response to asymmetric lattice conformational changes of inorganic enantiomers such as Te.^[^ ^31b^ ^]^ The high CD signal of 188 m^−1^deg^−1^ (Figure S11 Supporting Information) confirms the chirality‐guided growth for x = 0.5 at.% Sb content demonstrating the unique conformational variation due to the twisting lattice and atomic rearrangement.

**Figure 4:**
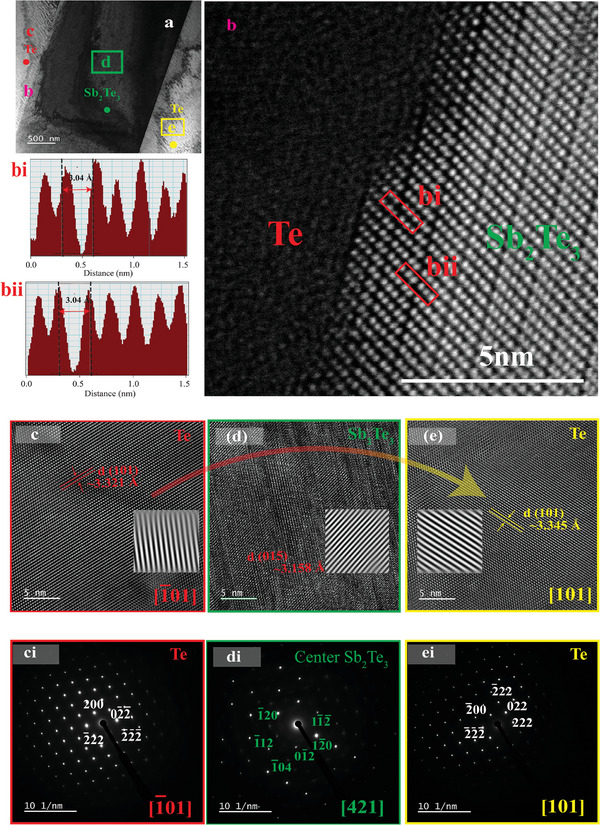
Characterization of Sb‐doped Te crystal (x = 1.5 at%) a) low magnified TEM of 1.5 at% Sb b) HAADF‐STEM of Te and Sb_2_Te_3_ phase boundary: (bi) and (bii) image intensity profiles of Te─Te interfaces of Sb_2_Te_3_ c) HRTEM of Te [$\bar{1}$01] d) HRTEM of Sb_2_Te_3_ [421] e) HRTEM of Te [101] (ci) SAED Te [$\bar{1}$01] (di) SAED Sb_2_Te_3_ [421] (ei) SAED Te [101].

Next, we correlated the influence of chiral twisting of the Te lattice as evidence of the chiral growth mechanism using 1.5 at% Sb ingots. The plate‐like microstructural characteristic is evident in the TEM image of temperature gradient solidification (TGS) ingot for 1.5 at% Sb in **Figure** **4**a and further complemented by focused ion beam‐scanning electron microscopy (FIB‐SEM) of thin sliced samples (Figure S12, Supporting Information). The representative HAADF STEM mapping and TEM‐EDS analysis (Figures S13 and S14, Supporting Information) shows only a minute amount of Sb dopants in the Te matrix which is consistent with our SEM analysis. Thus, large Sb_2_Te_3_ precipitates will be the main contributor to overall electronic conductivity. It is important to understand the structure‐property relationship on electrical transport properties. We observed from the HAADF‐STEM in Figure 4bi,bii that the interlayer value between each quintuple Te‐Sb‐Te‐Sb‐Te layer is ≈20% shorter than the theoretical van der Waals gap of Te─Te atoms. Moreover, the in situ atomic resolution of the Te─Te interphase of the Sb_2_Te_3_ precipitate and Te matrix is semicoherent with a d‐spacing of 3.2 Å which is equal to the interlayer spacing of the Sb_2_Te_3_ crystal.^[^ ^29a^ ^]^ This value is indicative of a significant interlayer coupling with modest charge transfer and sharing of electrons (0.244 e).^[^ ^40^ ^]^ From the HRTEM in Figure 4c,e the orientation of Te atoms at the extreme ends are non‐superimposable since they are oriented in the [$\bar{1}01]$ and [101] zone axis which is a characteristic of chiral materials. The corresponding SAED pattern in Figure 4ci–ei provides evidence that the Te has an asymmetric lattice about the planer Sb_2_Te_3_ phase boundary [421] zone axis. This observation presupposes that the Sb_2_Te_3_ achiral structure (space group; R $\bar{3}$ m) provides selective mediation of the conformal direction of atomic movements and reinforces chiral structure alignment but not the inherent chain chirality of Te. The geometric phase analysis from the HAADF‐STEM was used to determine the effects of strain at the atomically sharp interface between Sb_2_Te_3_ and Te (Figure S15, Supporting Information). The strain map ε_*xx*_ shows a prevalence of dislocation cores along the phase boundary which enhances phonon scattering. The lattice strains are created by misfit dislocations of Te─Te bonds estimated to be ≈0.11 Å. It is worth noting that most V_2_VI_3_ compounds like Sb_2_Te_3_ exhibit unconventional metavalent bonding from the low interatomic gap characteristics which lead to high electrical properties.^[^ ^25^, ^40^ ^]^ Hence in addition to the low grain boundary scattering in crystals, the modulated semicoherent interfacial effect provides electrically beneficial pathways for the chiral transport of charge carriers, and modest phonon propagation and hence an important consideration in thermoelectric properties.

### 2.4 Thermoelectric Transport Properties of Sb‐Doped Te

**Figure 5:**
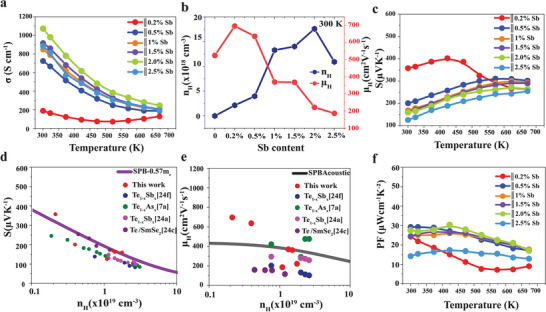
Thermoelectric properties of Sb‐doped Te crystals along the parallel growth direction. Temperature dependent a) electrical conductivity b) Hall carrier concentration and mobility at 300 K for varying compositions of Sb c) Seebeck coefficient d) Pisarenko plot where effective mass is calculated assuming single parabolic band model (SPB) against carrier concentration n_H_ e) mobility against carrier concentration f) temperature dependent power factor PF.

The anisotropic transport properties of pure Te ingot and spark plasma sintered sample are shown in (Figure S16, Supporting Information). A comparison of the transport properties along the perpendicular directions (Figure S17 Supporting Information) shows anisotropy of the Seebeck coefficient and electrical conductivity of ≈ *S*_∥_/*S*_⊥_ ≈ 1.26 and $\sigma_{∥}/\sigma_{⊥}∼1.4$, respectively as obtained by density functional theory (DFT) calculation.^[^ ^34^ ^]^ **Figure** **5**a, illustrates the degenerate behavior of electrical conductivity as a function of temperature. The electrical conductivity increases as the Sb content rises but declines when the Sb concentration exceeds 2.5 at. % Sb content. As anticipated, the Sb^3+^ ions contribute extra hole carriers, thereby increasing the carrier concentration from 2.08 × 10^−16^ cm^−3^ (pristine) to 1.45  × 10^19^ cm^−3^ for 1.5 at. % Sb as illustrated in Figure 5b. The high‐temperature Hall measurement corroborates the observed decrease in Hall mobility, due to high carrier concentration‐induced carrier–carrier scattering and additional scattering by Sb_2_Te_3_ precipitates (Figure S18, Supporting Information).

The Seebeck coefficient (Figure 5c) reveals a p‐type conductivity and exhibits degenerate semiconducting behavior due to the narrow bandgap of 0.34 eV estimated by Goldsmid–Sharp‘s relation.^[^ ^41^ ^]^ The density of states effective mass (m^*^≈0.57 m_e_) calculated using the single parabolic bands model,^[^ ^42^ ^]^ in Figure 5d is within the values (0.4–0.61 m_e_) obtained from polycrystalline samples.^[^ ^24^, ^43^ ^]^ The values of the Seebeck coefficient of the ingots are higher than those of the polycrystalline samples at similar carrier concentrations. The higher values are attributed to the effects of anisotropic transport properties which originated from asymmetric hole pockets of the Fermi surface as shown by DFT calculation along the c‐axis (Figure S19, Supporting Information).^[^ ^34^, ^44^ ^]^ Thus, the highly oriented ingots can exhibit a higher Seebeck coefficient than polycrystal samples due to their ideal electronic band structure characteristic. Sb doping does not induce any change in the band dispersion or predominant scattering mechanism by acoustic phonons µ_*H*,300*K*_ ≈ *T* ^−2.16^.^[^ ^24^, ^25^, ^45^ ^]^

The potential merit of Te and Sb_2_Te_3_ interfaces and low grain boundary scattering in maintaining high carrier mobility is emphasized in the single parabolic band (SPB) model Figure 5e. The temperature‐dependent transport properties of Sb‐doped Te are superior to Bi‐doped Te ingots (Figure S20, Supporting Information). Comparing the 0.5 at% doped samples as shown in (Figure S21 Supporting Information), the Hall mobility of Bi‐doped samples is ≈432 cm^2^ V^−1^ S^−1^ with a carrier concentration of ≈9.6 × 10^18 ^cm^−3^. However, Sb‐doped samples have high carrier mobility of ≈637 cm^2^ V^−1^ S^−1^ and carrier concentration of 3.96 × 10^18 ^cm^−3^. The carrier mobility deteriorates drastically with Bi doping. This is caused by the formation of highly resistive n‐type Bi_2_Te_3_ phase boundaries which increase carrier scattering in Te (Figure S22, Supporting Information). However, Sb_2_Te_3_ phase boundary precipitates always exhibit p‐type conductivity because of the low formation energy of antisite defects Sb_Te_ as such Sb_2_Te_3_ has less scattering effect on the electronic properties of Te.^[^ ^8c^ ^]^

Figure 5f shows a remarkably maximum power factor of ≈30 µWcm^−1^ *K* ^−2^ at 300 K and reaches ≈19 µWcm^−1^ *K* ^−2^ at 673 K for 0.5–2 at% Sb. This is because of enhanced high carrier concentration and low grain boundary scattering resulting in enhanced electrical conductivity and Seebeck coefficient. Moreover, the band alignment diagram in (Figure S23, Supporting Information) and interface potential according to the work functions of Te (4.95 eV) and Sb_2_Te_3_ (4.45 eV) is ≈0.26 eV. This low valence band offset implies that the Te and Sb_2_Te_3_ interfaces can concurrently maintain moderate carrier mobility by filtering low‐energy carriers. The calculated weighted mobility (µ_w_)^[^ ^37^ ^]^ (Figure S24a, Supporting Information) confirms the high‐power factor compared to polycrystalline Te.

### 2.5 Thermal Conductivity Behavior and High zT of Sb‐Doped Te Crystals

**Figure 6:**
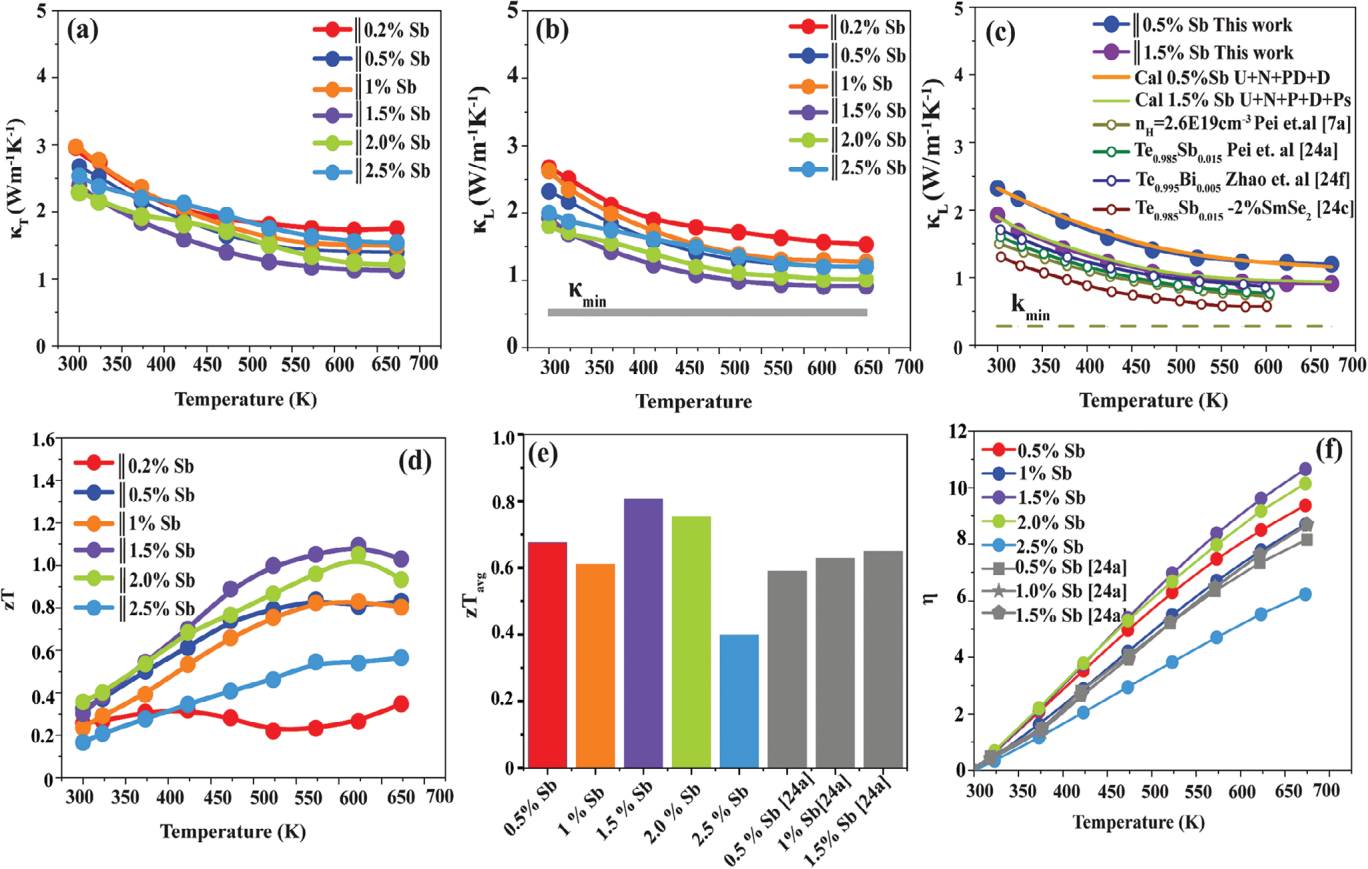
Thermal transport properties of Sb‐doped Te Temperature dependent a) Total thermal conductivity b) lattice thermal conductivity, the grey line shows the calculated minimum lattice thermal conductivity c) calculated Debye Callaway model with green lines denote the T^−1^ dependence because of Umklapp scattering, point defects and dislocation for 0.5 at% Sb‐doped Te, while orange lines include precipitate scattering to model lattice thermal conductivity of 1.5 at% Sb. In addition, a comparison is made with polycrystalline samples obtained from literature with similar composition d) figure of merit zT e) Average zT f) efficiency.

The temperature‐dependent total thermal conductivity decreases with Sb content as shown in **Figure** **6**a. The anisotropies at room temperature $\left(\kappa_{∥}/\kappa_{⊥}∼1.5\right)$ is comparable to single crystal Te $\left(K_{∥}/K_{⊥}∼2\right)$.^[^ ^46^ ^]^ It is known that the anisotropy is related to the contribution of lone pairs to structural anharmonicity as evidenced by the large Grüneisen parameter in the perpendicular direction.^[^ ^47^ ^]^ The electronic thermal conductivity is estimated by Wiedemann–Franz law(κ_*e*_ = *L_o_* σ*T*), and Lorenz number *L_o_* = 1.5 + *exp*[|*S*|/116]*W*Ω*K* ^−2^. The calculated lattice thermal conductivity in Figure 6b is higher than the theoretical value of κ_min_ = 0.28Wm^−1^K^−1^ estimated using the Cahill formula^[^ ^48^ ^]^ as confirmed by the measured transverse and longitudinal sound velocities in (Table S2, Supporting Information). The lattice thermal conductivity of Sb‐doped Te crystals is higher than the polycrystalline samples reported in the literature.^[^ ^24a,f^ ^]^ For polycrystal samples the low lattice thermal conductivity (≈1.8 Wm^−1^K^−1^) at 300 K decreases to (≈0.65 Wm^−1^K^−1^) at 670 K and is associated with polycrystalline grain boundary scattering with Sb_2_Te_3_ precipitate acting as an additional source of phonon scattering. However, in Sb‐doped crystal ingots the anisotropic lattice orientation introduces direction‐dependent thermal conductivity behavior reaching ≈3 Wm^−1^K^−1^ at 300 K. The lattice thermal conductivity (κ_latt_) of 1.5% Sb decreases significantly from ≈2.0 Wm^−1^K^−1^ at 300 K to ≈0.9 W m^−1^K^−1^ at 673 K (Figure S24b, Supporting Information). Despite the Sb_2_Te_3_ precipitates expected contribution to phonon scattering the highly oriented microstructure and low grain boundary scattering leads to high lattice thermal conductivity.

To quantitatively evaluate the contribution of Sb_2_Te_3_ precipitate scattering, the Debye–Callaway model was performed in Figure 6c assuming the Umklapp process and normal process $\left(\tau_{U+N}^{-1}\right)$ for scattering all frequency of phonons, boundary scattering $\left(\tau_{B}^{-1}\right)$, Dislocation core $\;(\tau_{DC}^{-1})$, Dislocation strain $\left(\tau_{DS}^{-1}\right)$ for mid‐frequency phonons scattering, point defect $\left(\tau_{PD}^{-1}\right)$ for low frequency scattering and precipitate scattering $\left(\tau_{Ps}^{-1}\right)$ by interfaces of Sb_2_Te_3_. As shown the phonon Umklapp scattering, and point defects are inadequate to describe the scattering behavior of 1.5% Sb. By, adding precipitate scattering the theoretical calculations κ_latt_ fit well with experimental values confirming the decrease in thermal conductivity.

The Sb‐doped Te crystal ingots prepared by the temperature gradient method have fewer grain boundaries than their counterpart polycrystals prepared using spark plasma sintering process, leading to high charge carrier transport and low grain boundary scattering. The power factor and high carrier mobility in elemental Te crystals compare favorably with other crystals grown by temperature gradient such as SnSe_2_,^[^ ^49^ ^]^ PbSe,^[^ ^50^ ^]^ AgPb_18+x_SbTe_20_,^[^ ^51^ ^]^ Mg_3_Sb_2_,^[^ ^52^ ^]^ PbSnS_2_,^[^ ^53^ ^]^ and GaTe^[^ ^54^ ^]^ as shown in (Figure S25, Supporting Information). However, the lattice thermal conductivity of crystals is higher than corresponding polycrystal samples. Thus, the simultaneous increase in power factor and decrease in thermal conductivity of 0.96 Wm^−1^K^−1^ for 1.5 at. % Sb leads to a high zT of 1.1 at 623 K in Figure 6d. Two crystals of similar composition were repeated in (Figure S26, Supporting Information). These values are remarkable since quasi‐ingot usually have higher thermal conductivities, however, the low grain boundary scattering enhances high carrier mobility eventually leading to a new high average zT value of 0.81 (Figure 6e) and high efficiency ≈10% (Figure 6f) for 1.5 at. % Sb‐doped Te compared with polycrystal samples.

## 3 Conclusion

This work shows that the thermoelectric performance of p‐type Te can be optimized by reducing grain boundary scattering via crystal growth. The Te crystals obtained by the temperature gradient method have fewer grain boundaries compared with polycrystals which leads to enhanced carrier mobility. The concomitant increase in carrier concentration and carrier mobility leads to a remarkable power factor of ≈30 µW cm^−1^K^−1^ in Te crystals. Second, we have unraveled that inclusions can mediate the lattice chirality in bulk Te crystals by forming semicoherent interfaces. Such interfaces are manifested by introducing Sb contents in the matrix which forms layered Sb_2_Te_3_ with Te─Te bonding to the matrix. The bonding heterogeneity and semicoherent interface between the Sb_2_Te_3_ rich phase and Te matrix act as possible sources of lattice rotation enabling an unusual chiral‐like twist mechanism of the lattice structure. In effect, the low grain boundary scattering constructs a pseudo‐fast atomic transport channel to maintain high carrier mobility paths. Because of the moderate electron‐phonon coupling by Sb_2_Te_3_ precipitates a remarkable decrease in lattice thermal conductivity was achieved. Consequently, a high zT_avg_ of 0.81, and corresponding efficiency of ≈10.1% were obtained. This work provides new insight into the mechanisms necessary for microstructural engineering to enhance transport properties.

## 4 Experimental Section

### Reagents and Materials

High‐purity commercial tellurium shots (99.99 wt.%, 5N Plus, Canada), Bi shots (99.99 wt.%, 5N Plus, Canada), and Sb (99.99 wt.%, 5N Plus, Canada) were used as starting materials.

### Synthesis‐Temperature Gradient Growth

The samples were weighed into a clean quartz tube ampoule. The ampoules were flame‐sealed after repeated cycles of purging with argon and loaded into an in‐house‐designed vertical furnace. The synthesis condition follows a slow heating rate of 5 ^○^ *C* min^−1^ to 510 ^○^ *C* maintained for 24 h and then slowly cooled to 330 °C at the rate of 1 °C*h* ^−1^. The sample was cooled to room temperature at 20 ^○^ *Ch* ^−1^. Te crystals with a diameter ≈15 mm and a weight of 50 g were cut into desired dimensions for TE characterization. For comparison, polycrystalline Te was synthesized using spark plasma sintering (SPS). Details of the synthesis condition are found in Figures S27 and S28 (Supporting Information).

### X‐Ray Diffraction Measurement

The powdered samples and bulk ingots were analyzed at room temperature using (SmartLab, Rigaku, Japan) with CuKα (λ = 1.5406 Å) radiation.

### X‐Ray Diffraction Pole figure

Texture analysis was performed using (D/Max 2500, Rigaku, Japan) in the parallel and perpendicular directions of the ingot.

### Raman Spectroscopy Measurements

The Raman spectra were obtained using high resolution/ PL system (LabRam HR Evolution Visible NR, Horiba) at room temperature.

### Scanning Electron Microscopy SEM Analysis

The Surface morphology and chemical compositions were analyzed using (FE‐SEM, HITACHI SU5000, Japan) at an acceleration voltage of 20 kV.

### Circular Dichroism Spectropolarimeter

The chiroptical response was measured by (J‐815, JASCO) equipped with a xenon lamp and photoelastic modulator, quad channel lock‐in amplifier.

### Suplarmolecular X‐ray Crystallography (XRC)

The single crystal X‐ray diffraction analyzer (Rigaku/XtaLABSynergy) with MoKα radiation (λ  =  0.71073 Å) was used to obtain the Laue diffraction pattern of the ingot.

### Transmission Electron Microscopy

For high‐resolution HR‐TEM the sample was prepared by focused ion beam technique (FIB: Helios G4, FEI Company, USA) with liquid Ga metal as an ion source. The final thickness of the sample was 40 nm after milling. The chiral structure and compositional analysis TEM‐EDS was observed using (cs‐STEM: Cs‐corrected HF5000 STEM/TEM, Hitachi, Japan) at 200 kV. The simulated atomic structure was performed with Vesta software. The microstructure of powdered samples was observed by Transmission electron microscopy (TEM) (Tecnai F30, FEI) with an operating voltage of 300 kV.

### Sound Velocity Measurement

The sound velocity in the parallel and perpendicular direction was measured by the pulse‐echo method. The transducer simultaneously acts as a source and receiver of the pulse signal. The ultrasonic pulse receiver (DPR 300, JSR Ultrasonic) in conjunction with a longitudinal transducer (CMR‐052, ndtXducer) and normal incidence shear transducer (SM‐052, ndtXducer) were used. The digital oscilloscope (DS0‐X 2022A, Keysight) was used to display the waveform. The time intervals from echoes were measured to determine the time delay of ultrasound reflections and calculated the speed of sound using the formula: ν = 2*d*/*t*, where t is the time delay and d is the sample thickness.

### Thermoelectric Property Measurements

The transport properties for Seebeck coefficient and electrical conductivity of machined samples of dimension (3 mm × 3 mm × 10 mm) were measured simultaneously by the commercial (ZEM‐3, ULVAC, Japan) samples under a continuous flow of He gas up to 673 K. The Thermal conductivity of the samples (8 mm × 8 mm × 2 mm) was measured using a laser flash technique (LFA 467, Netzsch Ltd, Germany). The thermal conductivity was determined by the general formula κ = ρCpD, where density (ρ) was obtained from the geometric dimension and weighted mass, C_p_ is the specific heat capacity estimated by the Dulong–Petit approximation and D is diffusivity. Density was determined by Archimedes principle. The high‐temperature Hall measurement system (ResiTest 8300, TOYO Corporation, Japan) was measured from 300–673 K. The below room temperature Hall measurement was performed with (ECOPIA HMS 3000).

### Density Functional Theory (DFT)

The Seebeck coefficient and Fermi surface were calculated with the electronics module implemented in the MedeA software environment, which uses the Vienna ab initio simulation package (VASP) to estimate the electronic structure.^[^ ^55^ ^]^ A cutoff energy of 400 eV and a 23  ×  23  ×  16 k‐mesh were used to obtain the transport properties and Fermi surface.

## Conflict of Interest

The authors declare no conflict of interest.

## Supporting information

Supporting Information

## Data Availability

The data that support the findings of this study are available from the corresponding author upon reasonable request.

## References

[1] a) G. J. Snyder , E. S. Toberer , Nat. Mater. 2008, 7, 105; 18219332 10.1038/nmat2090

[2] a) C. L. Chen , T. H. Wang , Z. G. Yu , Y. Hutabalian , R. K. Vankayala , C. C. Chen , W. P. Hsieh , H. T. Jeng , D. H. Wei , Y. Y. Chen , Adv. Sci. 2022, 9, 2201353; 10.1002/advs.202201353 PMC9284191 35478495

[3] a) H. Cho , S. Y. Back , J. H. Yun , S. Byeon , H. Jin , J.‐S. Rhyee , ACS Appl. Mater. Interfaces 2020, 12, 38076; 32805971 10.1021/acsami.0c09529

[4] a) D. Feng , Z.‐H. Ge , D. Wu , Y.‐X. Chen , T. Wu , J. Li , J. He , Phys. Chem. Chem. Phys. 2016, 18, 31821; 27841409 10.1039/c6cp06466c

[5] a) J. P. Heremans , B. Wiendlocha , A. M. Chamoire , Energy Environ. Sci. 2012, 5, 5510;

[6] a) S. Zulkifal , Z. Wang , X. Zhang , S. Siddique , Y. Yu , C. Wang , Y. Gong , S. Li , D. Li , Y. Zhang , Adv. Sci. 2023, 10, 2206342; 10.1002/advs.202206342 PMC10265067 37092577

[7] a) S. Lin , W. Li , Z. Chen , J. Shen , B. Ge , Y. Pei , Nat. Commun. 2016, 7, 10287; 26751919 10.1038/ncomms10287 PMC4729895

[8] a) H. Jang , M. Y. Toriyama , S. Abbey , B. Frimpong , J. P. Male , G. J. Snyder , Y. S. Jung , M. W. Oh , Adv. Mater. 2022, 34, 2204132; 10.1002/adma.202204132 35944565

[9] a) L. Abdellaoui , Z. Chen , Y. Yu , T. Luo , R. Hanus , T. Schwarz , R. Bueno Villoro , O. Cojocaru‐Mirédin , G. J. Snyder , D. Raabe , Adv. Funct. Mater. 2021, 31, 2101214;

[10] a) S. Chandra , U. Bhat , P. Dutta , A. Bhardwaj , R. Datta , K. Biswas , Adv. Mater. 2022, 34, 2203725; 10.1002/adma.202203725 36028167

[11] a) K. Biswas , J. He , I. D. Blum , C.‐I. Wu , T. P. Hogan , D. N. Seidman , V. P. Dravid , M. G. Kanatzidis , Nature 2012, 489, 414; 22996556 10.1038/nature11439

[12] Y. Pan , M. Yao , X. Hong , Y. Zhu , F. Fan , K. Imasato , Y. He , C. Hess , J. Fink , J. Yang , Energy Environ. Sci. 2020, 13, 1717.

[13] R. K. Vankayala , T. W. Lan , P. Parajuli , F. Liu , R. Rao , S. H. Yu , T. L. Hung , C. H. Lee , S. i. Yano , C. R. Hsing , Adv. Sci. 2020, 7, 2002494. 10.1002/advs.202002494 PMC7740100 33344133

[14] A. T. Duong , V. Q. Nguyen , G. Duvjir , V. T. Duong , S. Kwon , J. Y. Song , J. K. Lee , J. E. Lee , S. Park , T. Min , Nat. Commun. 2016, 7, 13713. 27941762 10.1038/ncomms13713 PMC5160008

[15] W.‐D. Liu , L.‐C. Yin , L. Li , Q. Yang , D.‐Z. Wang , M. Li , X.‐L. Shi , Q. Liu , Y. Bai , I. Gentle , Energy Environ. Sci. 2023, 16, 5123.

[16] Q. Deng , F. Zhang , P. Nan , Z. Zhang , L. Gan , Z. Chen , B. Ge , H. Dong , H. k. Mao , R. Ang , Adv. Funct. Mater. 2024, 34, 2310073.

[17] W. Wu , C. Zhu , H. Ming , T. Chen , D. Li , X. Qin , J. Zhang , Nanoscale 2022, 14, 17163. 36374160 10.1039/d2nr04419f

[18] X. Zhong , H. Zhou , C. Li , A. G. Shtukenberg , M. D. Ward , B. Kahr , Chem. Commun. 2021, 57, 5538. 10.1039/d1cc01431e 33960341

[19] M. Sakano , M. Hirayama , T. Takahashi , S. Akebi , M. Nakayama , K. Kuroda , K. Taguchi , T. Yoshikawa , K. Miyamoto , T. Okuda , Phys. Rev. Lett. 2020, 124, 136404. 32302163 10.1103/PhysRevLett.124.136404

[20] S. S. Tsirkin , P. A. Puente , I. Souza , Phys. Rev. B 2018, 97, 035158.

[21] Y. Liu , W. Wu , W. A. Goddard III, J. Am. Chem. Soc. 2018, 140, 550. 29268604 10.1021/jacs.7b09964

[22] a) T. Fukuroi , S. Tanuma , S. Tobisawa , Sci. Rep. Res. Inst. Ser. A 1949, 1, 373;

[23] T. Fukuroi , S. Tanuma , S. Tobisawa , Sci. Rep. Res. Inst. Ser. A 1952, 4, 283.

[24] a) S. Lin , W. Li , X. Zhang , J. Li , Z. Chen , Y. Pei , Inorg. Chem. Front. 2017, 4, 1066;

[25] a) D. An , S. Zhang , X. Zhai , W. Yang , R. Wu , H. Zhang , W. Fan , W. Wang , S. Chen , O. Cojocaru‐Mirédin , Nat. Commun. 2024, 15, 3177; 38609361 10.1038/s41467-024-47578-w PMC11014947

[26] S. Abbey , H. Jang , B. Frimpong , N. Kumar , W. H. Nam , J. H. Park , C. V. Nguyen , H. Shin , J. Y. Song , S.‐D. Park , Energy Environ. Sci. 2023, 16, 125.

[27] a) I. Dierking , Liq. Cryst. 2001, 28, 165;

[28] L. Fu , K. H. Lee , S.‐I. Kim , J.‐H. Lim , W. Choi , Y. Cheng , M.‐W. Oh , Y.‐M. Kim , S. W. Kim , Acta Mater. 2021, 215, 117058.

[29] a) H. Mun , K. H. Lee , S. J. Yoo , H.‐S. Kim , J. Jeong , S. H. Oh , G. J. Snyder , Y. H. Lee , Y.‐M. Kim , S. W. Kim , Acta Mater. 2018, 159, 266;

[30] A. Londono‐Calderon , D. J. Williams , M. M. Schneider , B. H. Savitzky , C. Ophus , S. Ma , H. Zhu , M. T. Pettes , Nanoscale 2021, 13, 9606. 34002755 10.1039/d1nr01442k

[31] a) C. Li , A. G. Shtukenberg , D. J. Carter , X. Cui , I. Olson , A. L. Rohl , J. D. Gale , P. Raiteri , B. Kahr , J. Phys. Chem. C 2018, 122, 25085;

[32] B. Sung , A. de La Cotte , E. Grelet , Nat. Commun. 2018, 9, 1405. 29643349 10.1038/s41467-018-03745-4 PMC5895742

[33] S. Ibaraki , R. Ise , K. Ishimori , Y. Oaki , G. Sazaki , E. Yokoyama , K. Tsukamoto , H. Imai , Chem. Commun. 2015, 51, 8516. 10.1039/c5cc01466b 25892326

[34] H. Peng , N. Kioussis , G. J. Snyder , Phys. Rev. B 2014, 89, 195206.

[35] J. J. Kuo , S. D. Kang , K. Imasato , H. Tamaki , S. Ohno , T. Kanno , G. J. Snyder , Energy Environ. Sci. 2018, 11, 429.

[36] C. Hu , Z. Gao , M. Zhang , S. Han , C. Fu , T. Zhu , Energy Environ. Sci. 2023, 16, 5381.

[37] G. J. Snyder , A. H. Snyder , M. Wood , R. Gurunathan , B. H. Snyder , C. Niu , Adv. Mater. 2020, 32, 2001537. 10.1002/adma.202001537 32410214

[38] X. Zhang , Z. Bu , X. Shi , Z. Chen , S. Lin , B. Shan , M. Wood , A. H. Snyder , L. Chen , G. J. Snyder , Sci. Adv. 2020, 6, eabc0726. 33188018 10.1126/sciadv.abc0726 PMC7673762

[39] Z. Dong , Y. Ma , Nat. Commun. 2020, 11, 1588. 32221297 10.1038/s41467-020-15388-5 PMC7101389

[40] Y. Cheng , O. Cojocaru‐Mirédin , J. Keutgen , Y. Yu , M. Küpers , M. Schumacher , P. Golub , J. Y. Raty , R. Dronskowski , M. Wuttig , Adv. Mater. 2019, 31, 1904316. 10.1002/adma.201904316 31489721

[41] Z. M. Gibbs , H.‐S. Kim , H. Wang , G. J. Snyder , Appl. Phys. Lett. 2015, 106, 022112.

[42] J. Zhu , X. Zhang , M. Guo , J. Li , J. Hu , S. Cai , W. Cai , Y. Zhang , J. Sui , npj Comput. Mater. 2021, 7, 116.

[43] M. Yang , X. Li , S. Duan , X. Zhang , H. Sun , X. Chen , T. Su , L. Gu , X. Liu , Adv. Energy Mater. 2022, 12, 2203014.

[44] a) M.‐W. Oh , J.‐J. Gu , H. Inui , M.‐H. Oh , D.‐M. Wee , Phys. B 2007, 389, 367;

[45] M. Yang , T. Su , S. Li , S. Li , M. Hu , X. Liu , J. Alloys Compd. 2021, 887, 161342.

[46] A. Adams , F. Baumann , J. Stuke , Phys Status Solidi A Appl Mater Sci 1967, 23, K99.

[47] H. Peng , N. Kioussis , D. A. Stewart , Appl. Phys. Lett. 2015, 107, 251904.

[48] D. G. Cahill , S. K. Watson , R. O. Pohl , Phys. Rev. B 1992, 46, 6131. 10.1103/physrevb.46.6131 10002297

[49] a) C. Li , W. He , D. Wang , L.‐D. Zhao , Chin. Phys. B 2021, 30, 067101;

[50] S. Wang , Y. Wen , Y. Zhu , Z. Wang , D. Liu , J. Zheng , S. Zhan , H. Xie , Z. Ge , X. Gao , Small 2024, 2400866, 10.1002/smll.202400866. 38639306

[51] Y. Zhu , Y. Yu , H. Zhang , Y. Qin , Z.‐Y. Wang , S. Zhan , D. Liu , N. Lin , Y. Tao , T. Hong , J. Am. Chem. Soc. 2023, 145, 24931. 10.1021/jacs.3c09655 37922502

[52] a) K. Imasato , C. Fu , Y. Pan , M. Wood , J. J. Kuo , C. Felser , G. J. Snyder , Adv. Mater. 2020, 32, 1908218; 10.1002/adma.201908218 32115799

[53] S. Zhan , T. Hong , B. Qin , Y. Zhu , X. Feng , L. Su , H. Shi , H. Liang , Q. Zhang , X. Gao , Nat. Commun. 2022, 13, 5937. 36209153 10.1038/s41467-022-33684-0 PMC9547848

[54] T. H. Vu , A. T. Pham , J. Park , S. Park , S. Cho , J. Solid State Chem. 2021, 298, 122155.

[55] a) G. Kresse , D. Joubert , Phys. Rev. B 1999, 59, 1758;

