# Supplementary material for: Chiral Twist Interface Modulation Enhances Thermoelectric Properties of Tellurium Crystal
Source: Adv Sci (Weinh). 2024 Jul 23;11(35):2402147. doi: 10.1002/advs.202402147 (PMC11425292; doi:10.1002/advs.202402147)
Supplement: Supplementary file 1 — Supporting Information [file ADVS-11-2402147-s001.docx]

Supporting Information

**Chiral Twist Interface Modulation Enhances Thermoelectric Properties of Tellurium Crystal**

*Stanley Abbey, Hanhwi Jang, Brakowaa Frimpong, Van Quang Nguyen, Jong Ho Park, Su-Dong Park, Sunglae Cho, Yeon Sik Jung, Ki-Ha Hong, and Min-Wook Oh**


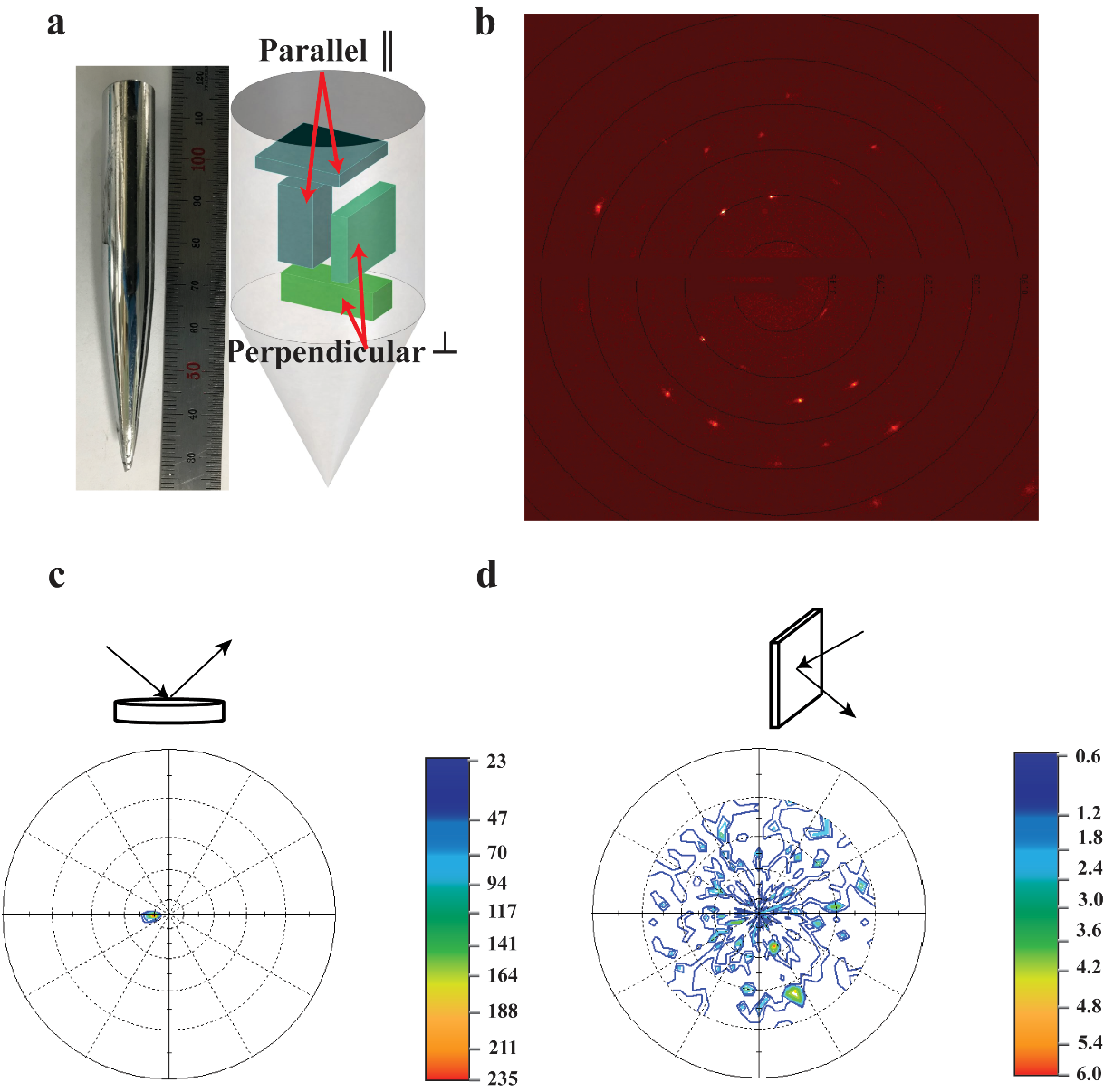


**Figure S1**. (a) Synthesized Te crystal ingot obtained by temperature gradient solidification with anisotropic measurement directions. (b) Laue diffraction pattern of Sb doped Te (x=0.005) crystal. (c) XRD pole figure in parallel direction (d) XRD pole figure in the perpendicular direction.

**Supplementary note**

In Figure S2a the Te lattice structure has a weak interchain Van der Waals (vdW) interaction (d_NNN_) in the ab-axis and covalent intrachain (d_NN_) bonding along the c-axis (001). The typical hexagonal Moiré superlattice is observed in Figure S2b confirming the relative twist of atomic layers along the (001) due to screw dislocations mediated growth. The Raman spectrum shows highest peak at 121 cm^-1^ for A^1^ mode associated with chain expansion in the ab plane while the degenerate E^1^ (92 cm^-1^) and E^2^ (140 cm^-1^) is related to bond bending in the a-axis and bond stretching c-axis, respectively.


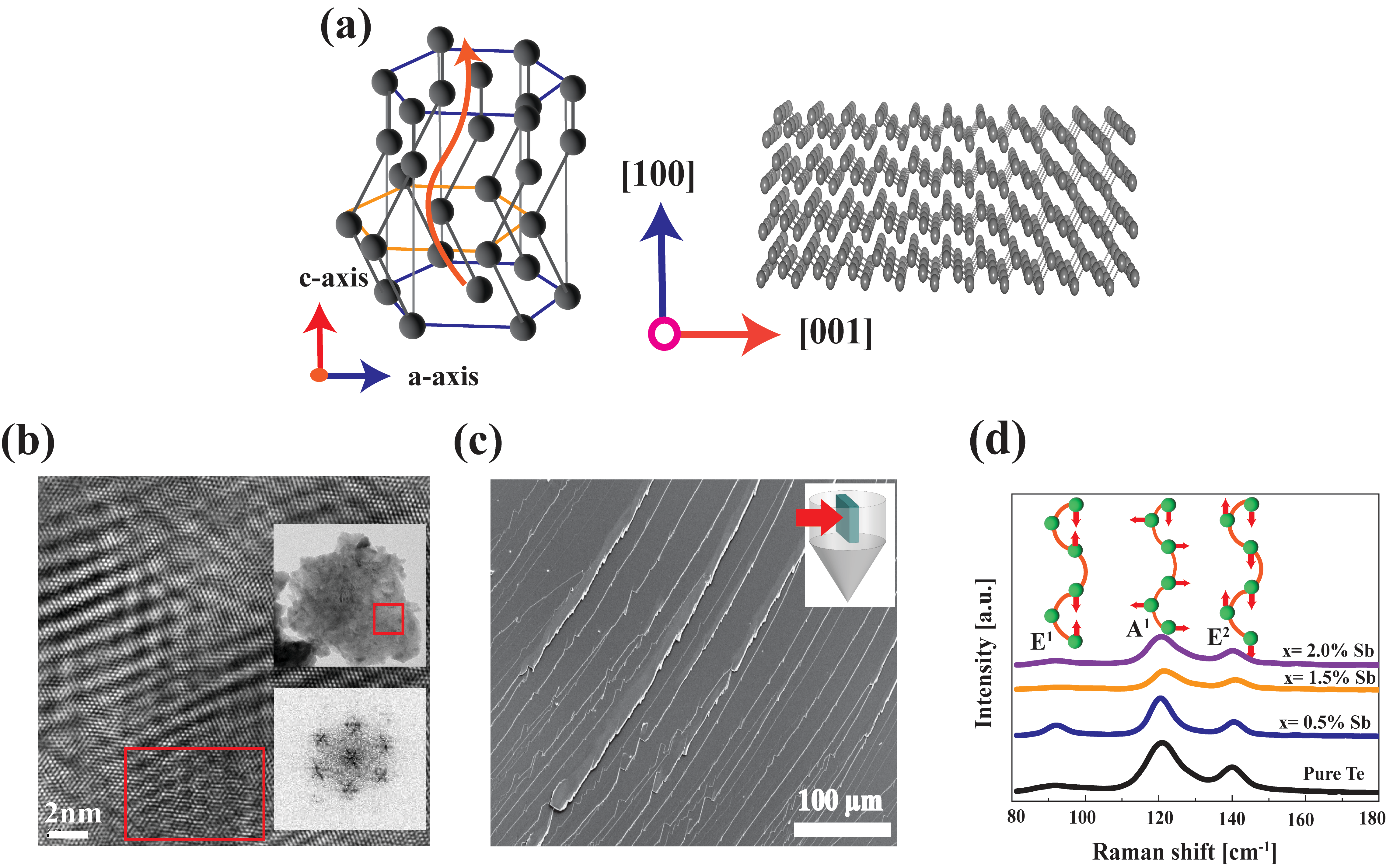


**Figure S2**. **Crystal structure of Te**. (a) the anisotropic hexagonal lattice of Te with atoms at the corners projected along the (001) with Chiral atomic structure (b) HRTEM of powdered Sb doped Te showing a Moiré pattern (red box) along projected; the inset FFT (c) Fracture surface tellurium ingot showing layered plates (d) Raman spectrum of Sb doped Te samples


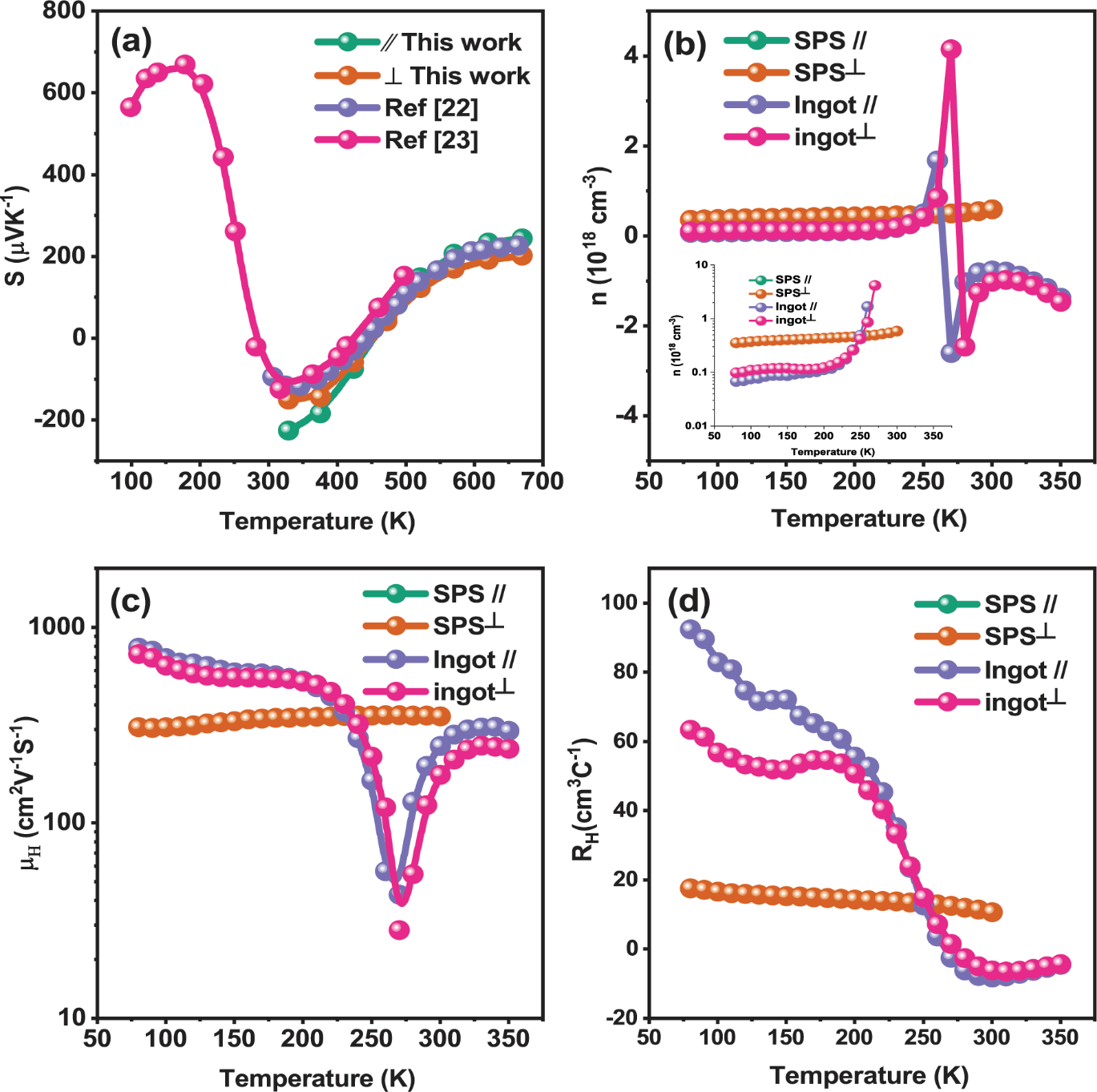


**Figure S3**. **Low temperature hall measurement in the parallel** (⫽) and perpendicular (┴) for pureTe (a) comparing the conductivity switching p-n-p Seebeck coefficient of pure Te (b) Hall carrier concentration (n_H_) the inset corresponds to the log scale to differentiate the carrier concentration of ingots and SPS (c) Hall mobility μ_H_ (d) average Hall coefficient (R_H_).


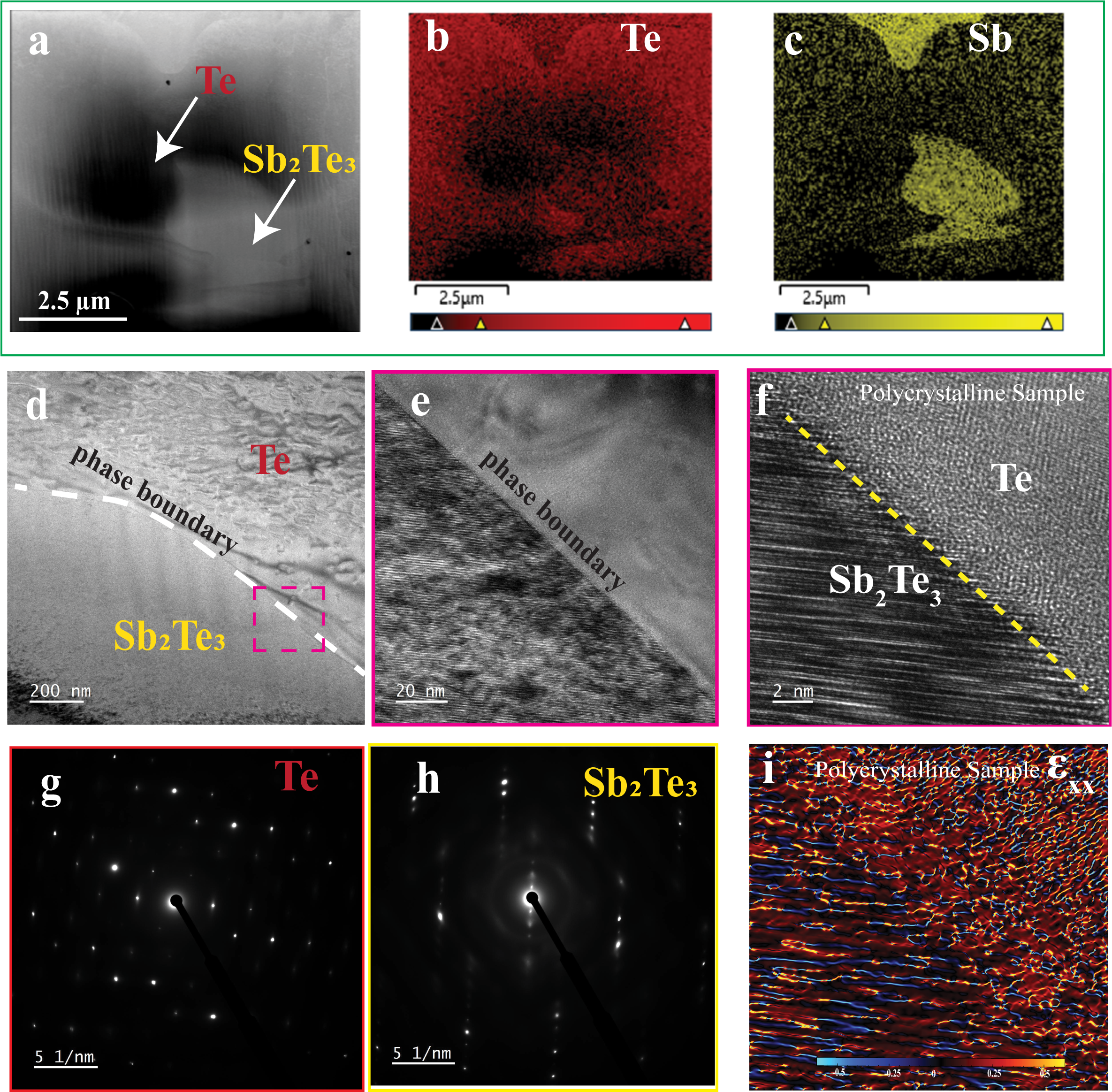


**Figure S4**.Transmission electron microscopy (TEM) image of the Sb_2_Te_3_ precipiate in Te matrix obtained by spark plasma sintering(SPS).(a) low magnification TEM (b,c) TEM-EDS of the composition (d,e, f) phase boundary interface (g,h) Selected area electron diffraction (SAED) pattern of the matrix Te(red) and Sb_2_Te_3_ (yellow), repectively.(i) GPA strain anylysis of polycrytalline sample.


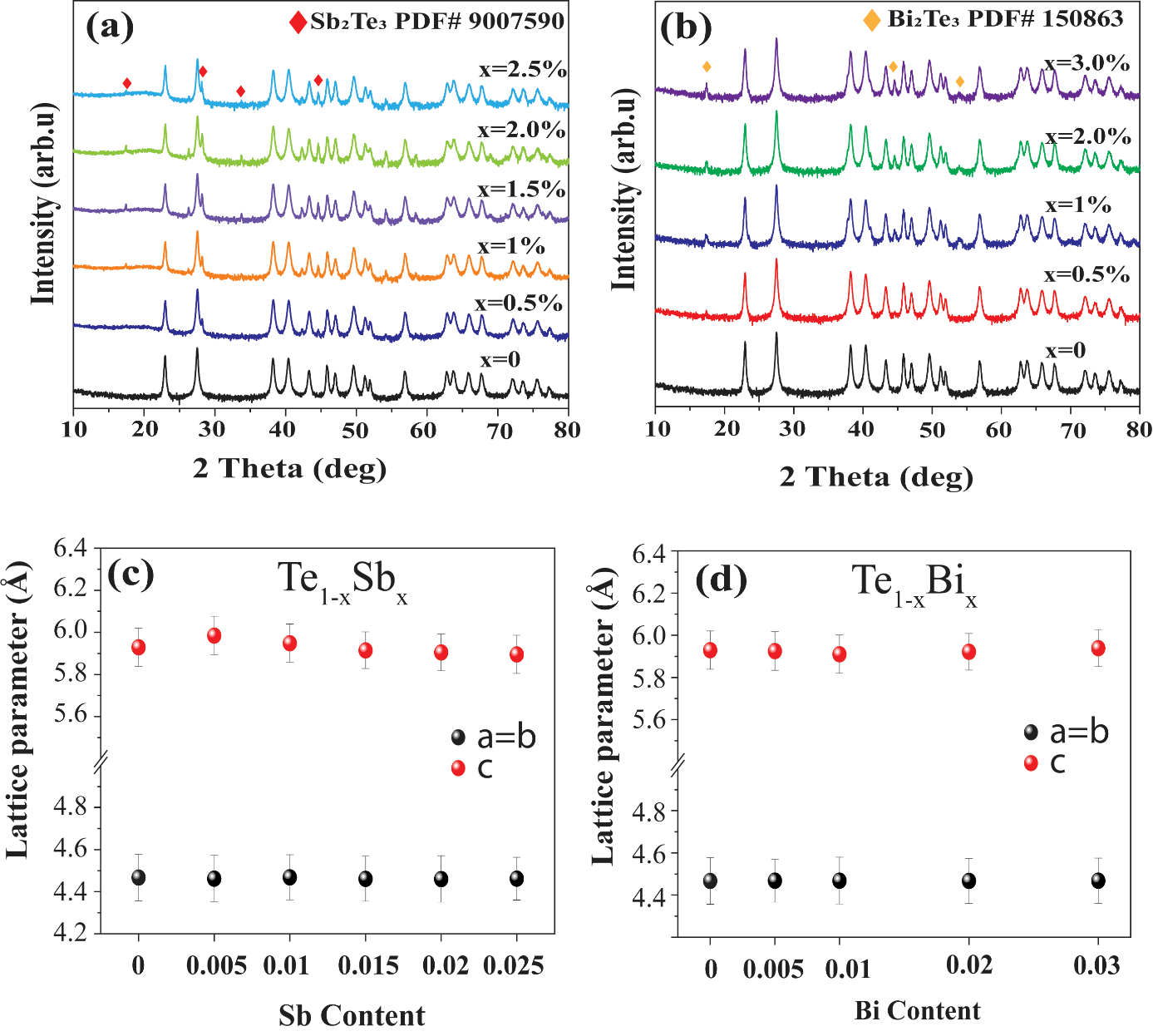


**Figure S5**. Powdered XRD (log scale of intensity) (a) Sb doped Te (red dot-Sb_2_Te_3_) (b) Bi doped Te (yellow dot Bi_2_Te_3_). Calculated lattice parameter (c) Sb doped Te (d) Bi doped Te.

**Table S1.** SEM-EDX point analysis of samples. The results indicate the formation of Sb_2_Te_3_ in the matrix of Te.

|  | **Mole ratio (%)** | | |
| --- | --- | --- | --- |
| **Sample** | **Sb** | **Te** | **SEM-EDX** |
| **X=1.0 % Sb** | 38.48 | 61.52. | 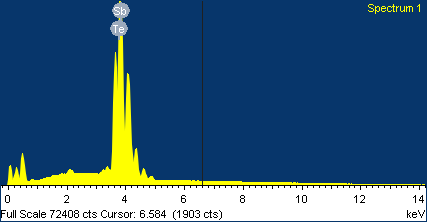 |
| **X=1.5 % Sb** | 39.65 | 60.35 | 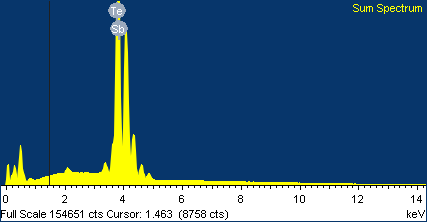 |
| **X=2.0 % Sb** | 39.50 | 60.50 | 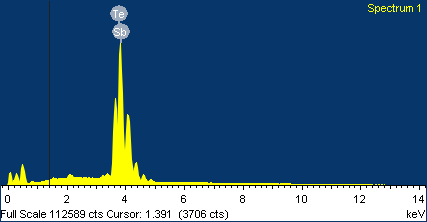 |
| **X=2.5 % Sb** | 39.88 | 60.12 | 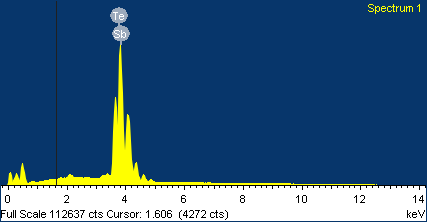 |
|  |  |  |  |


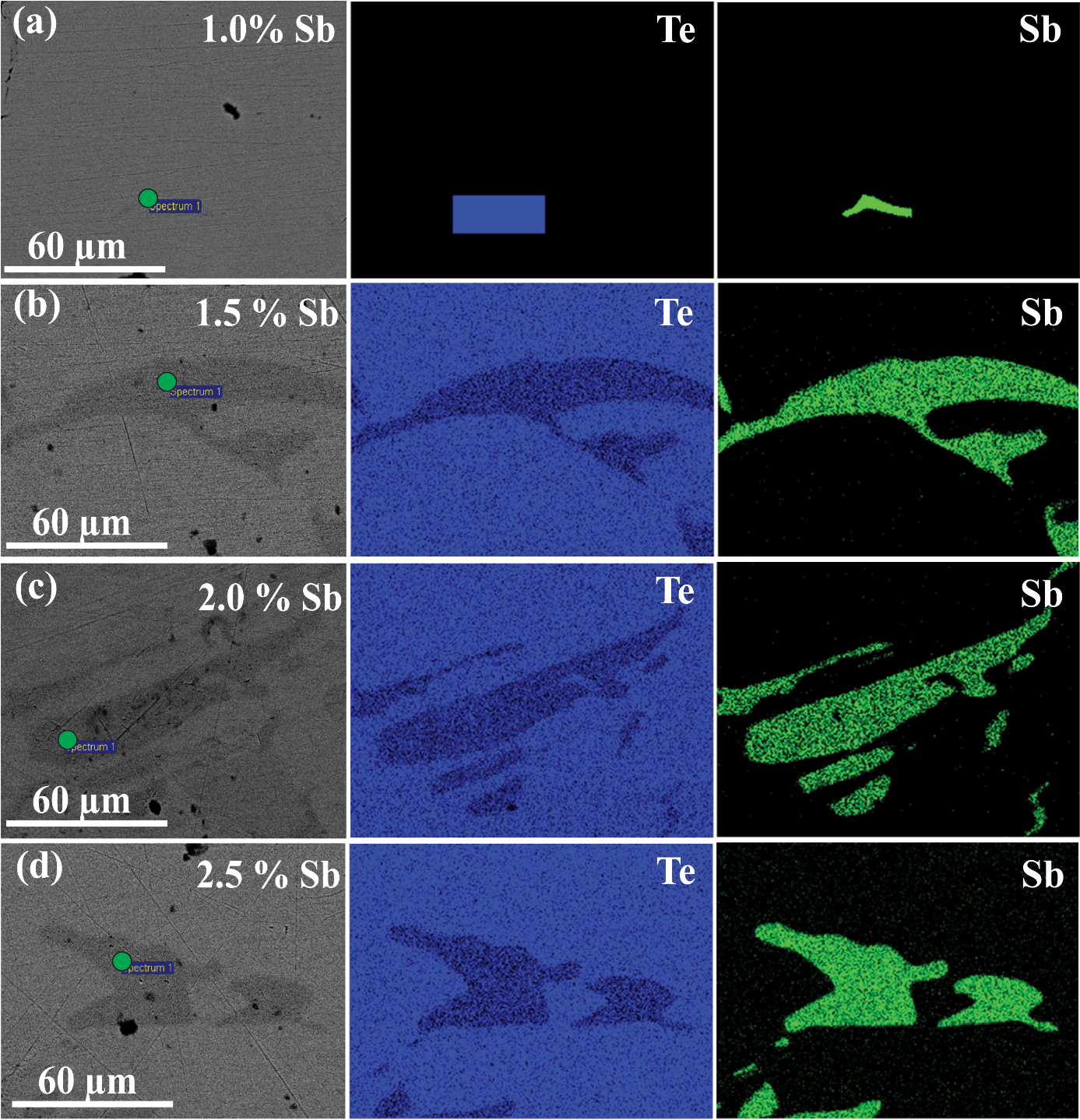


**Figure S6. Scanning electron microscopy (SEM- EDX)** (a-d) Sb doped Te at various compositions. The blue and green color represents Te and the secondary phases of Sb_2_Te_3_, respectively.


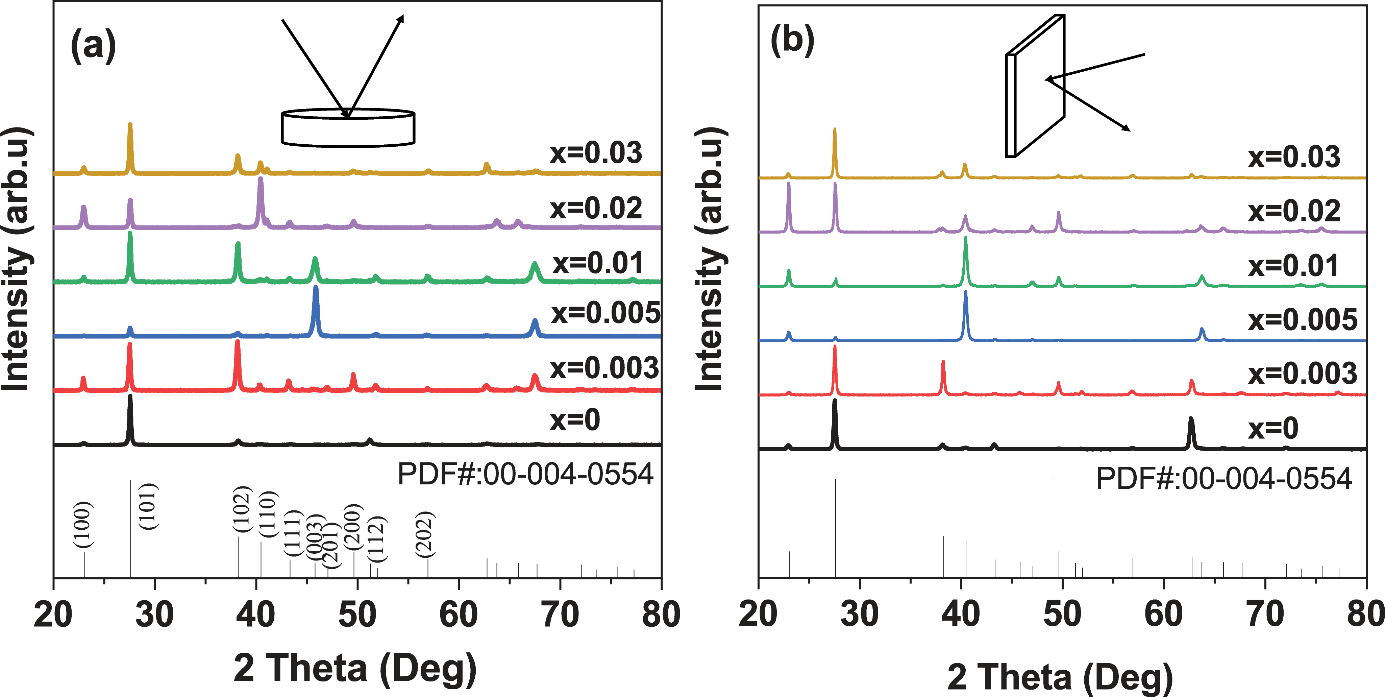


**Figure S7**. XRD profile of Bi doped Te ingot (a) perpendicular direction (┴) (b) parallel (⫽) to the growth direction.


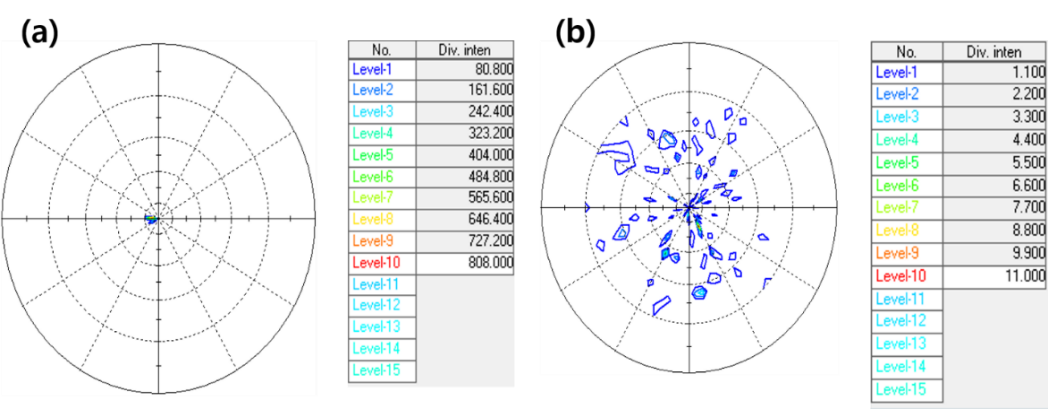


**Figure S8**. XRD profile of Bi doped Te ingot (x=0.5%) (a) perpendicular direction (┴) (b) parallel (⫽) to the growth direction.


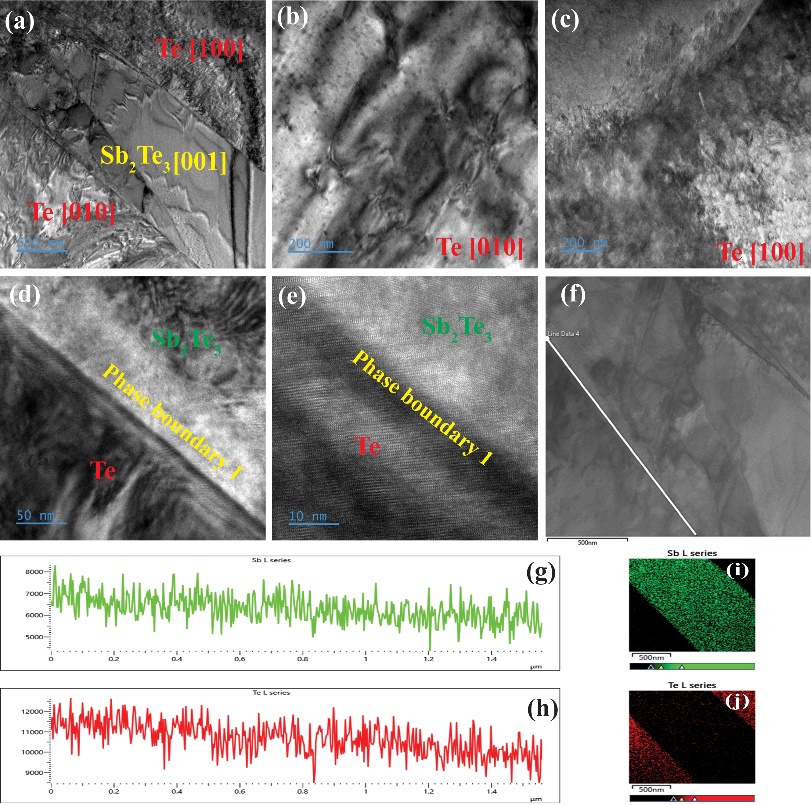


**Figure S9.** (a) Low magnification TEM characterization of 0.5% ingot (b) Te [010] (c) Te [100]. (d, e) HRTEM along phase boundary 1 (f, g, h) TEM-EDX line scan along the collinear interface (i, j) TEM-EDX mapping of Sb and Te


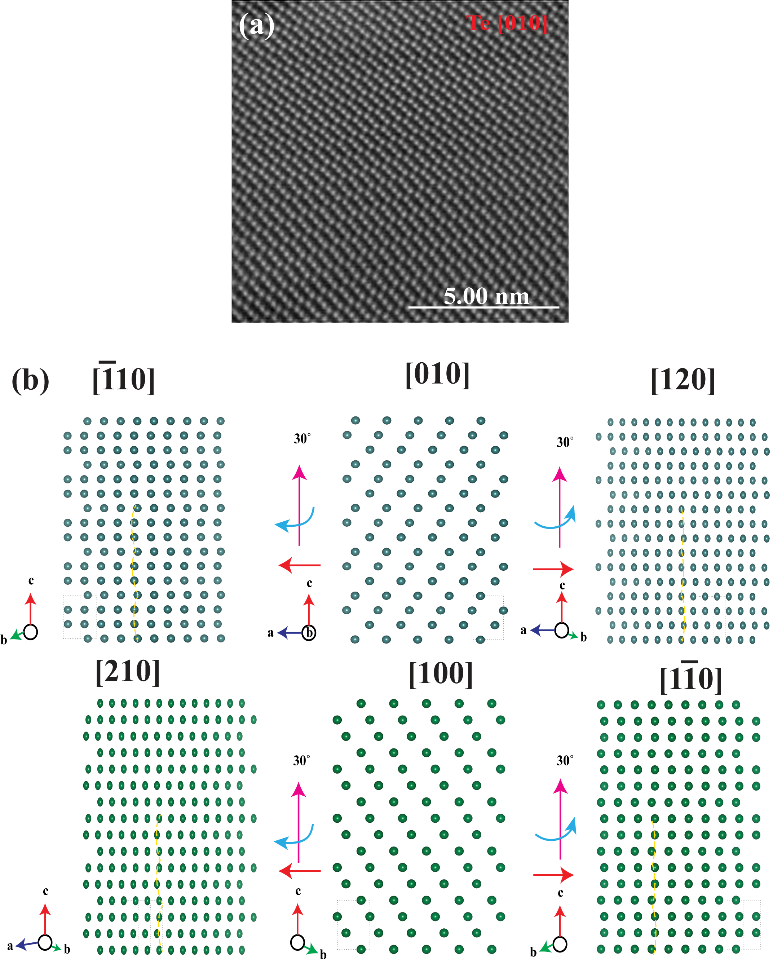


**Figure S10.** (a) STEM characterization of 0.5% ingot mapping in [010] (b) Tilt series of chiral atoms of Te.


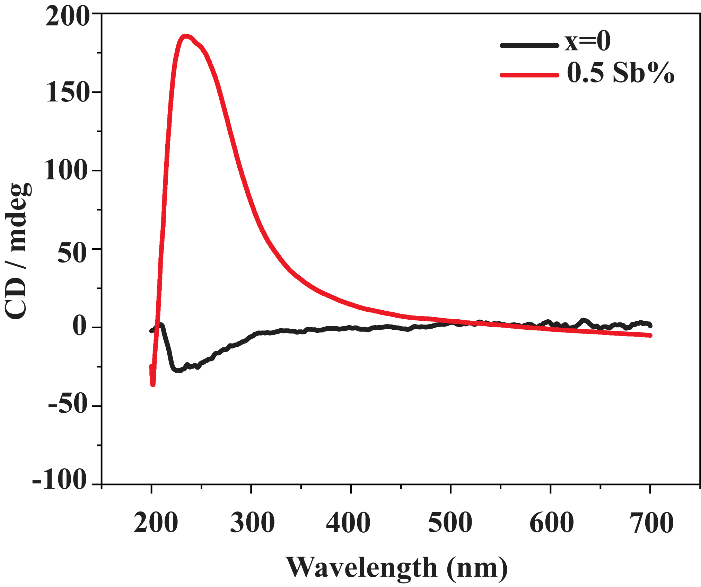


**Figure S11** Circular dichroism of Te-0.5 at% Sb ingots.


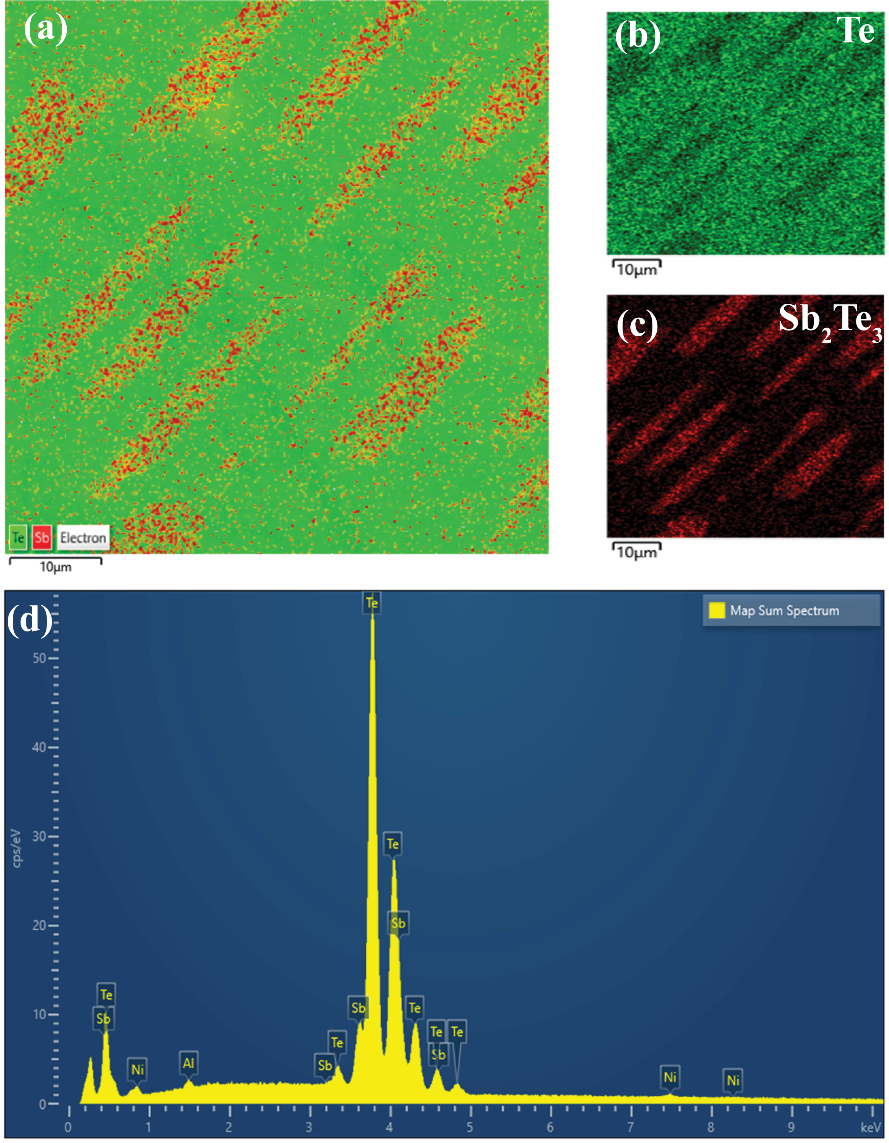


**Figure S12. FIB-Sliced TEM samples for 1.5% Sb** (a) EDS map (b) Te (c) Sb (d) EDS spectrum with small additional peaks of Ni Kα, Al Kα from chamber


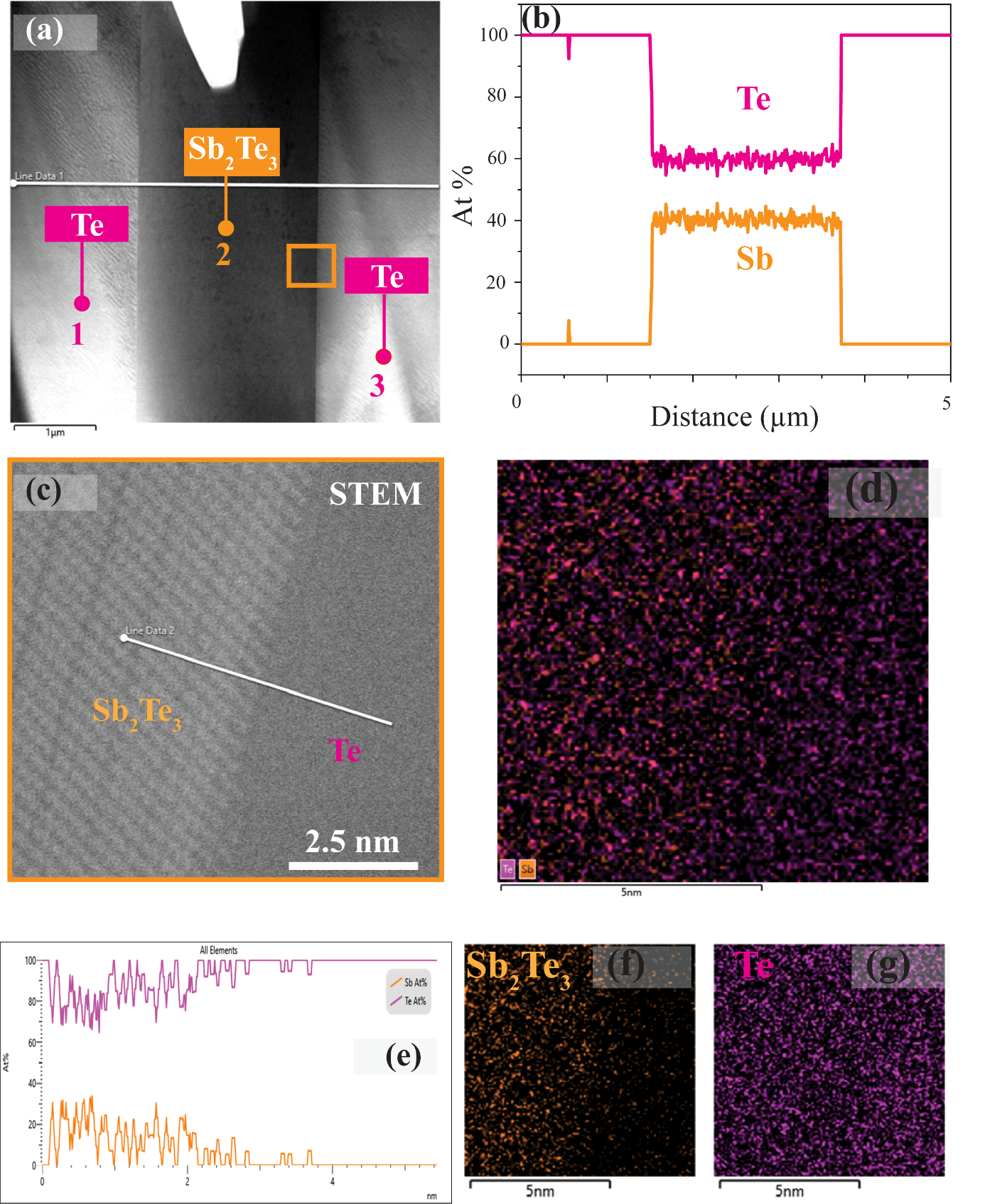


**Figure S13**. (a) Low magnification TEM (b) TEM-EDS across the Te-Sb-Te (C) HAADF-STEM image at the interface (marked as square) between Te and Sb_2_Te_3_ (d) EDS mapping of interface (e) TEM-EDS Line profile spectrum along interface (f) Sb_2_Te_3_ (g) Te


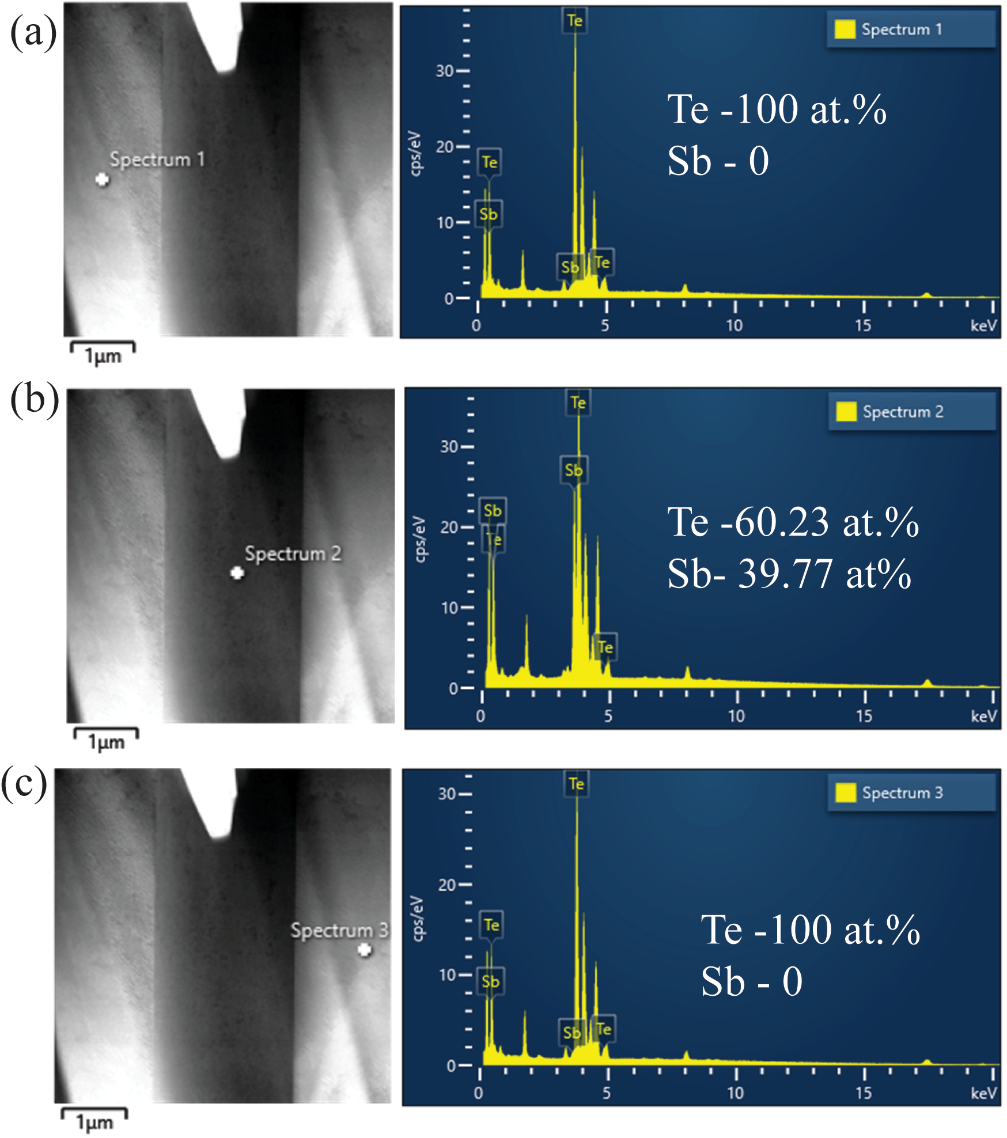


**Figure S14**. (a-c) TEM-EDX point analysis.


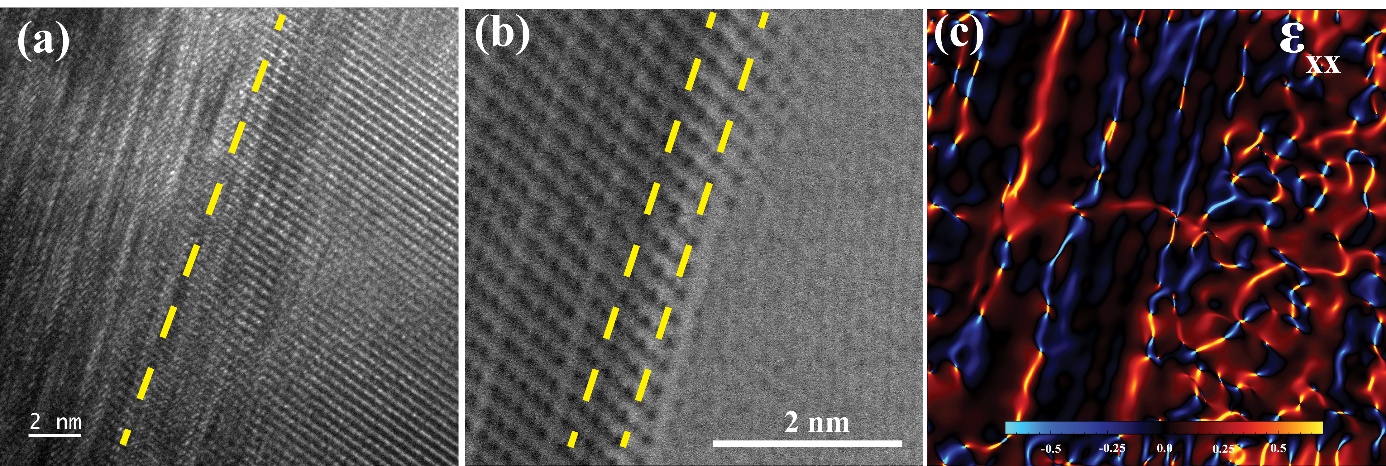


**Figure S15.** (a) HRTEM along the interface (b) HAADF STEM at the interface (c) Geometric phase analysis (GPA) between Sb_2_Te_3_ and Te region.


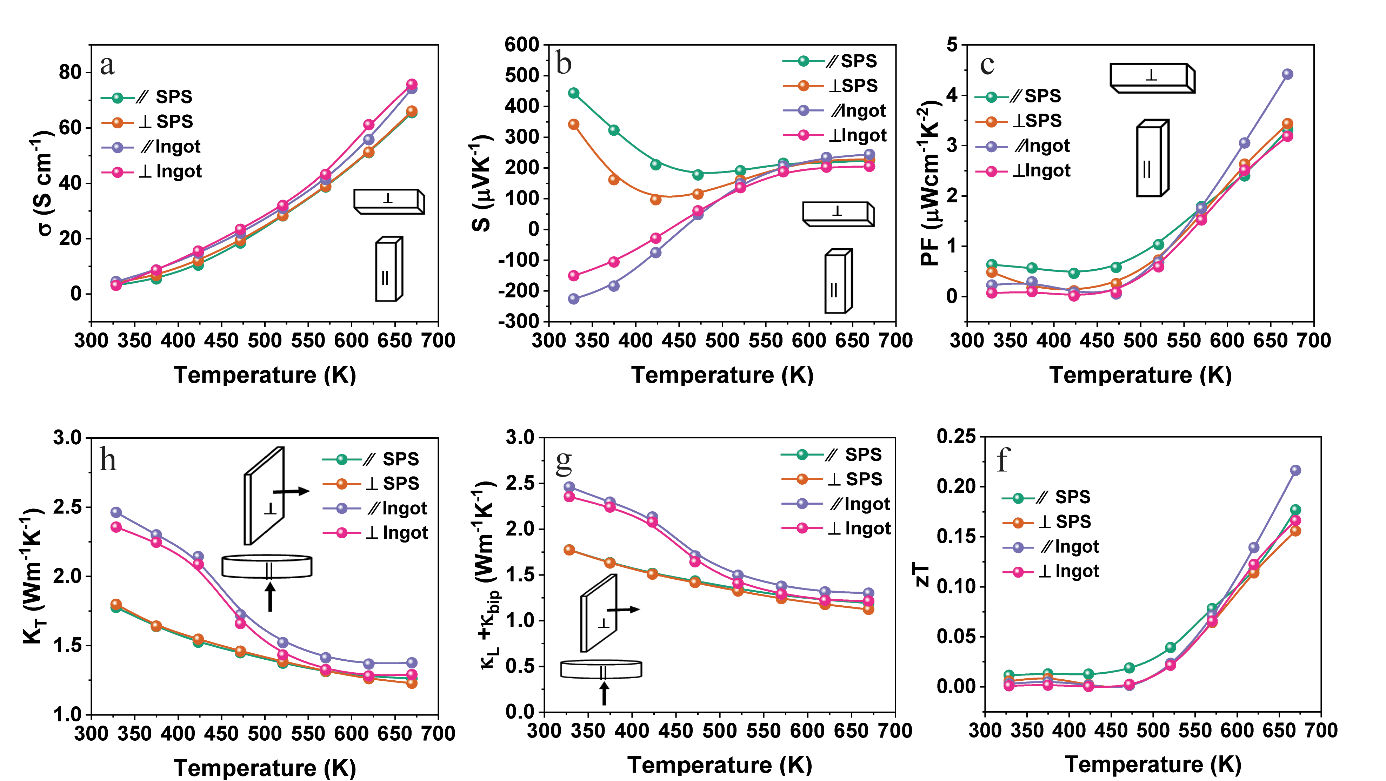


**Figure S16**. **Thermoelectric Transport properties of pure Te crystal**. Temperature-dependent thermoelectric properties of spark plasma sintered (SPS) and TGS ingots of pure Te in parallel (⫽) and perpendicular (┴) direction (a) electrical conductivity (b) Seebeck coefficient (c) power factor (d) Total thermal conductivity (e) lattice thermal conductivity (f) figure of merit zT.

Pure Te shows anomalous semiconducting behavior with increasing temperature which is prevalent in single crystals. Electrical conductivity indicates non-degenerate properties with increase temperature Figure (S17a). The n-p-n transition in Seebeck coefficient Figure (S17b) is calculated elsewhere by relaxation time approximation. The intrinsic bipolar conduction and low carrier concentration results in a low power factor (Figure S17c) and relatively high total thermal and lattice thermal conductivity (Figure S17 d, e) leading to a moderate zT of 0.21 at 623 K in Figure S17e.


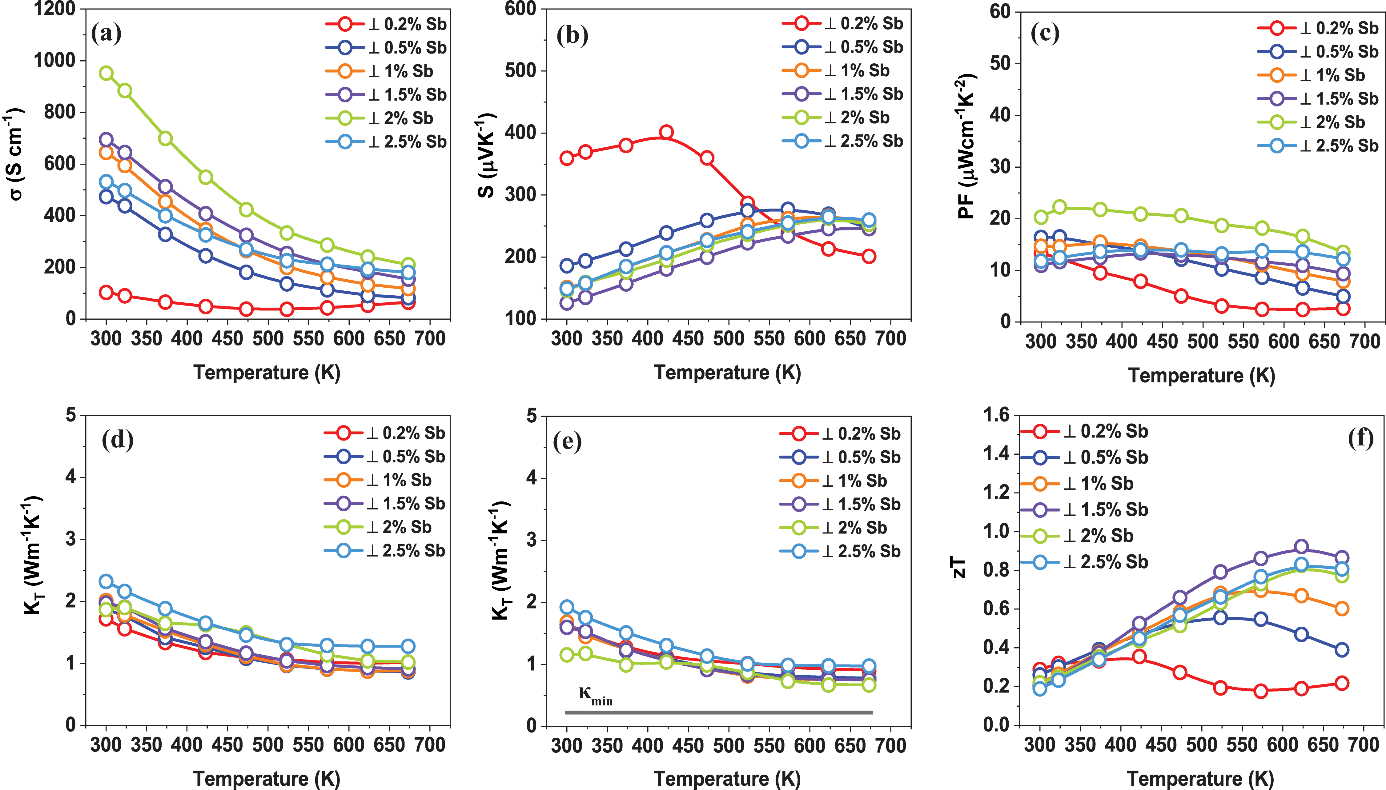


**Figure S17. Transport properties** (a) Electrical conductivity (b) Seebeck coefficient (c) power factor (d) total thermal conductivity (e) lattice thermal conductivity (f) Figure of merit, zT.

**Table S2.** Sound velocity measurement in parallel and perpendicular direction at 300 K.

| **sample** | **V_L_(m/s)** | **V_t_(m/s)** | **V_m_(m/s)** |
| --- | --- | --- | --- |
| Sb-0.2% | 3419.2 | 1417.2 | 1603.25 |
| Sb-0.5% | 3546.88 | 2202.3 | 2427.47 |
| Sb-1% | 3430.2 | 1865.1 | 2080.577 |
| Sb-1.5% | 3324.3 | 1652.2 | 1853.88 |
| Sb-2.0% | 3377.15 | 1758.5 | 2567.8 |
| Sb-2.5% | 3482.5 | 1809.5 | 2646.1 |

**
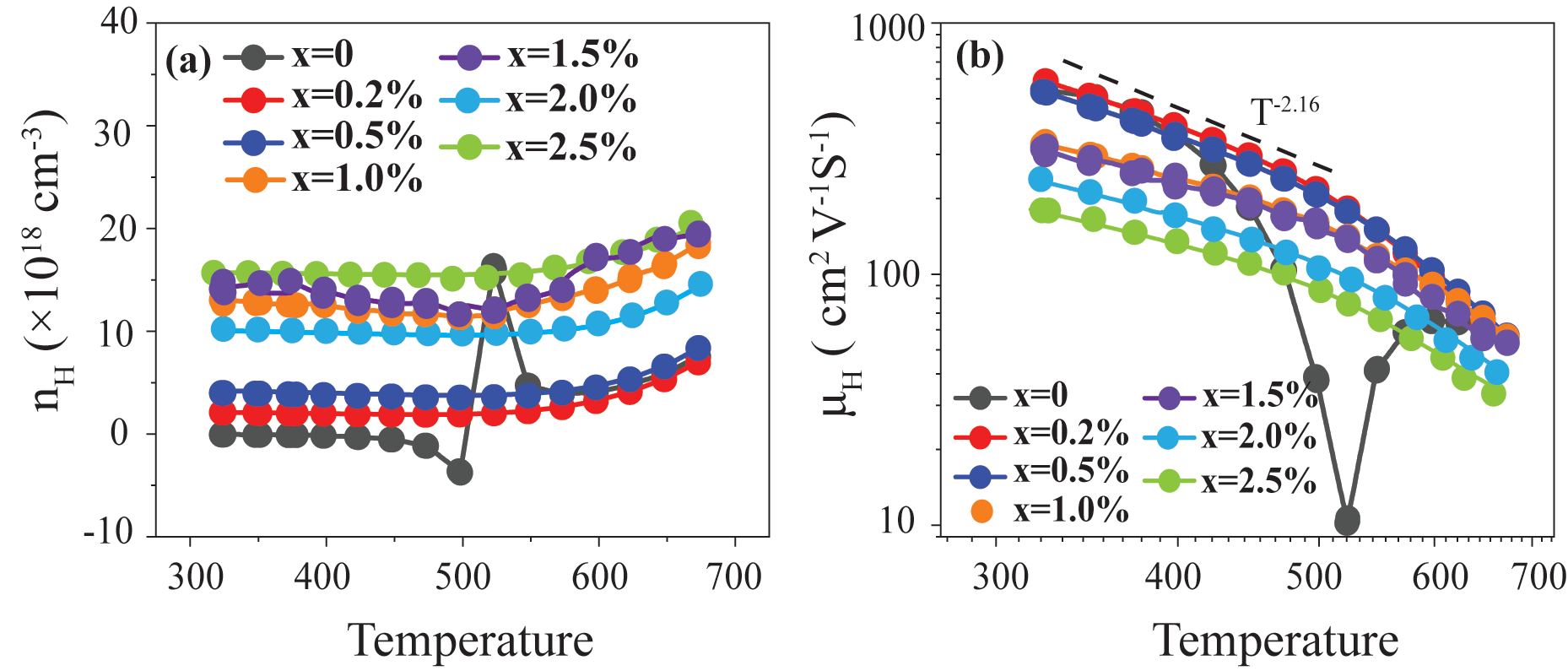
**

**Figure S18**. (a) High temperature dependent Hall carrier concentration (b) Hall mobility.


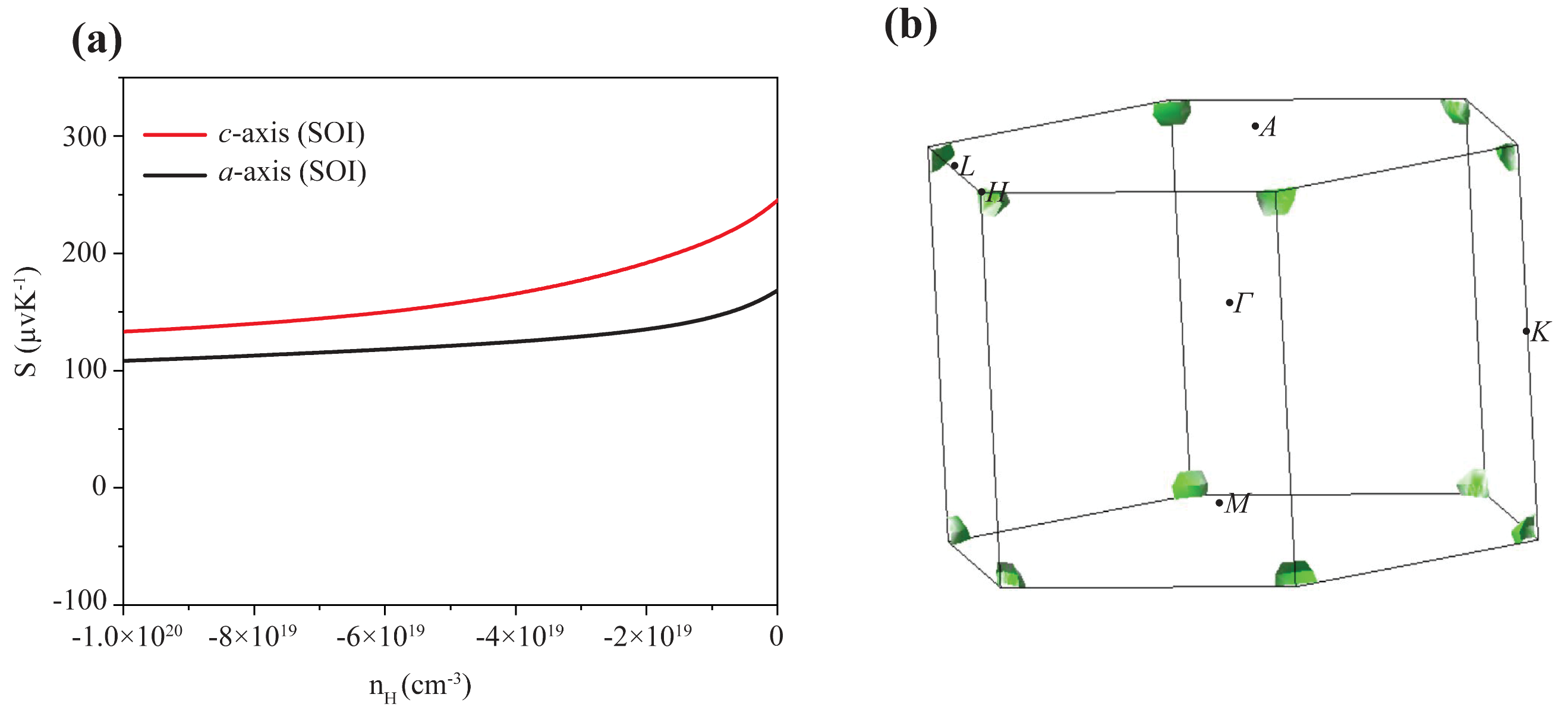


**Figure S19. Results from density functional theory (DFT) calculations.** (a) Calculated Seebeck coefficient with spin-orbit interaction as a function of carrier concentration along the c-axis (red line) and a-axis (black line) of Te. (b)The Fermi surface in Brillouin zone. The surface was obtained at -50 meV from the valence band maximum.


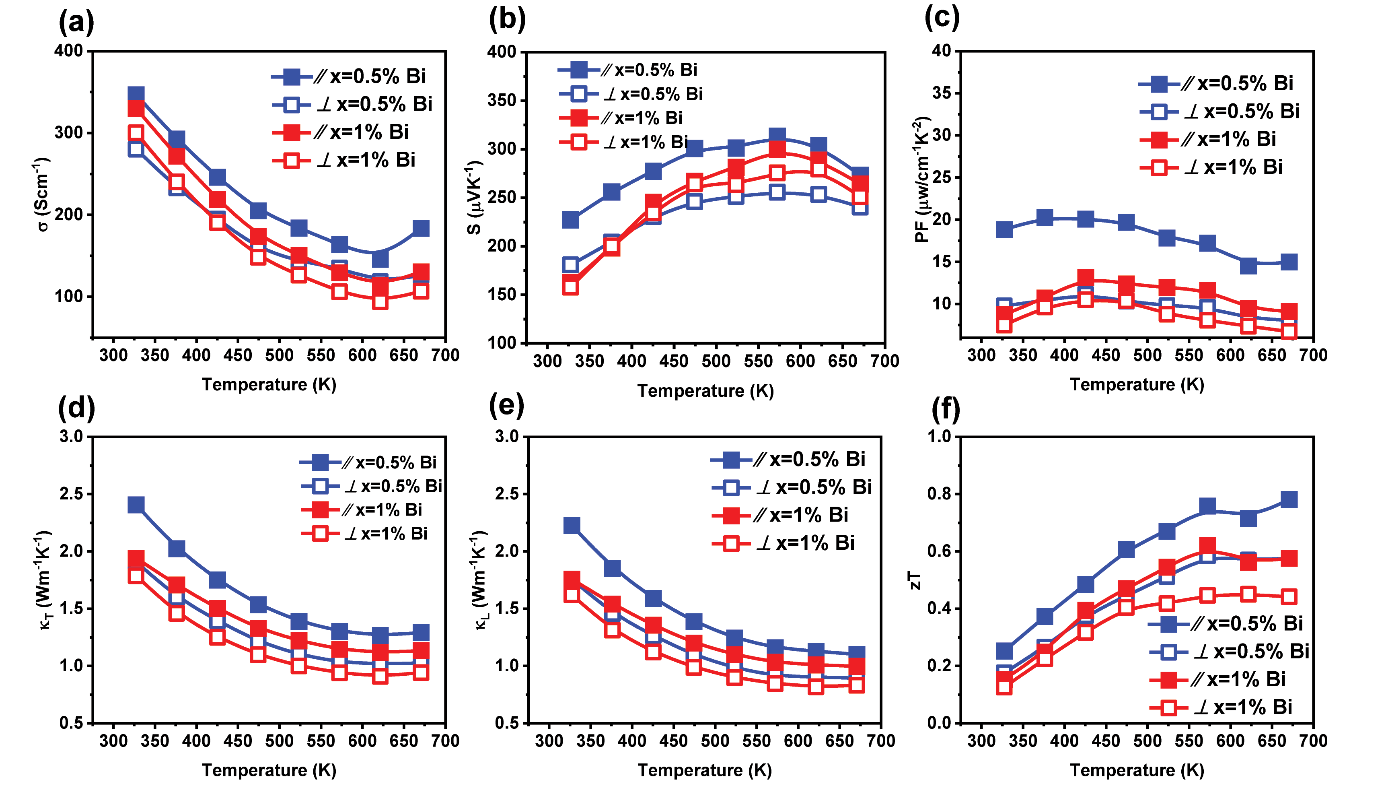


**Figure S20.** Thermoelectric properties in the parallel (⫽) and perpendicular (┴) direction for Te_1-x_(Bi)_x_ (x=0, 0.5,1at%). (a) electrical conductivity (b) Seebeck coefficient (c) power factor. (d) total thermal conductivity Bi and Sb (e) lattice thermal conductivity Bi (f) figure of merit, zT.


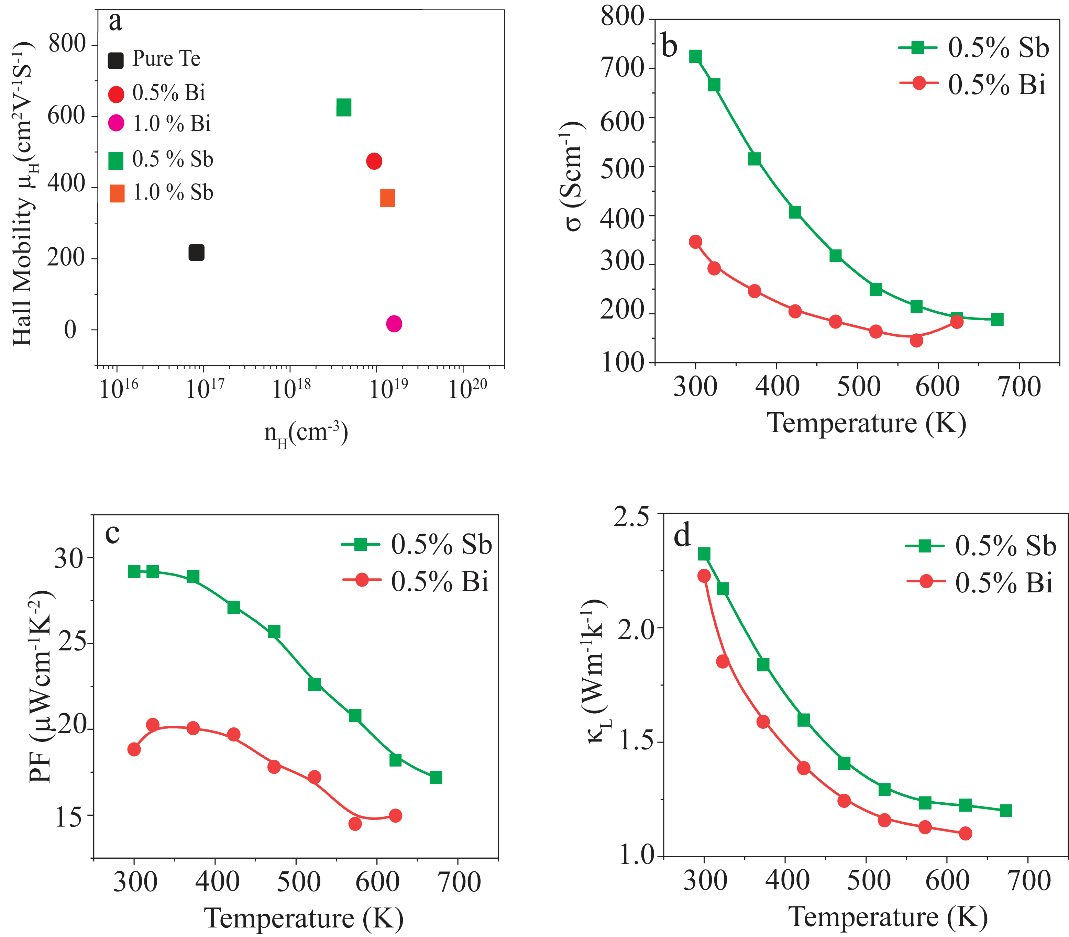


**Figure S21**.Comparing transport properties of 0.5 at.% Bi and Sb doped Te (a) Room temperature dependent Hall carrier concentration and Hall mobility (b) Electrical conductivity (b) Power factor (c) Lattice thermal conductivity.


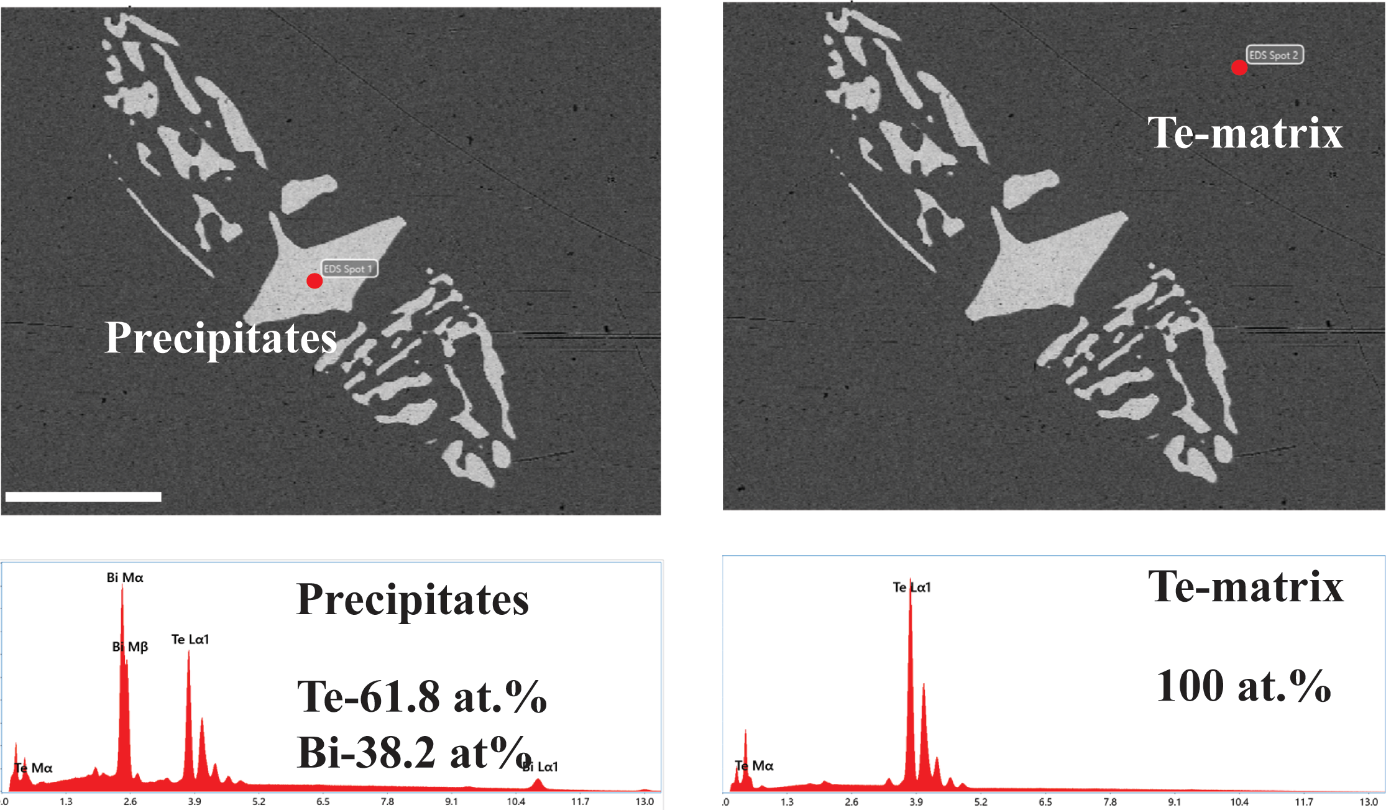


**Figure S22**. SEM-EDS of 0.5 % doped Te


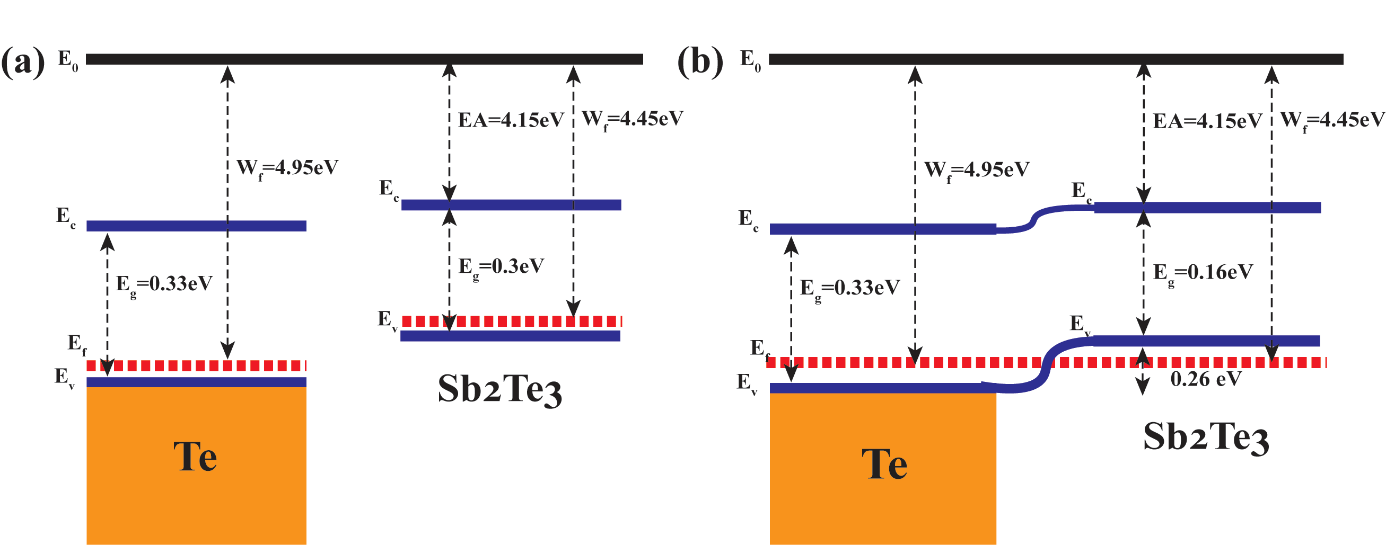


**Figure S23.** (a, b) Band diagram for Te and Sb_2_Te_3._ Source code for determination of valence band maximum, conduction band minimum and Fermi level from materials project database.


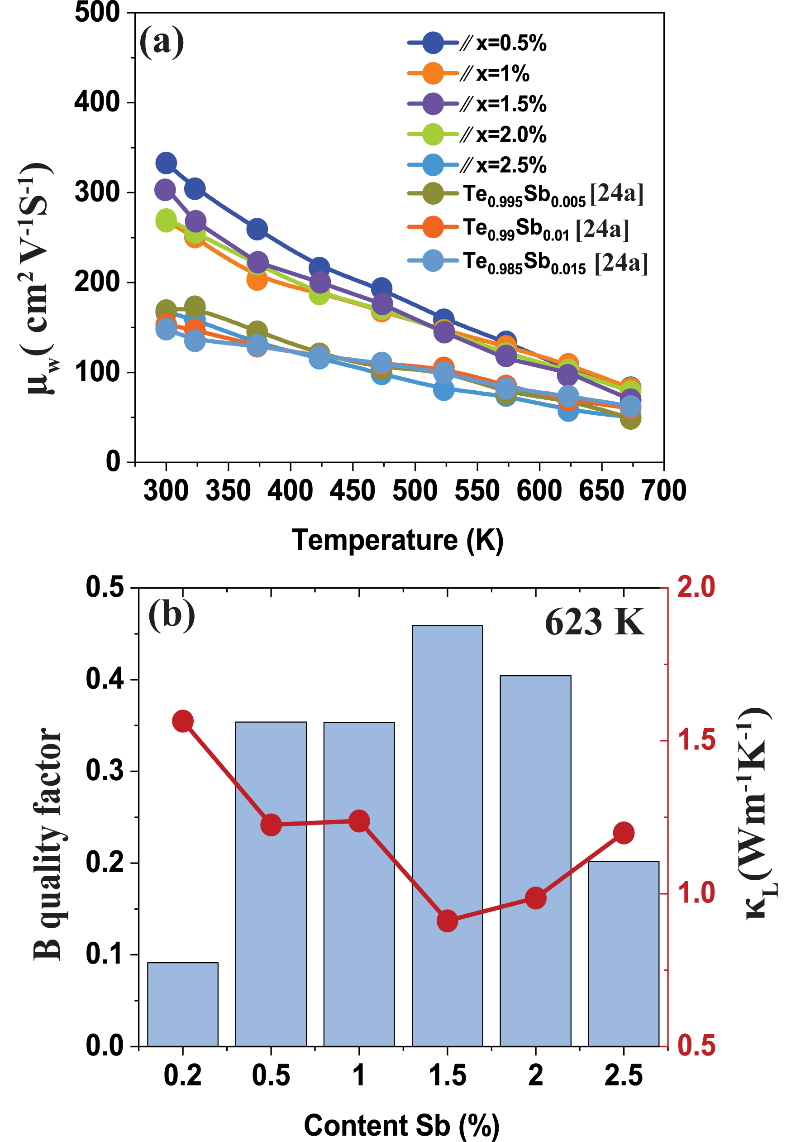


**Figure S24.** Calculated weighted mobility (a) Sb doped Te (b) Material quality factor B.


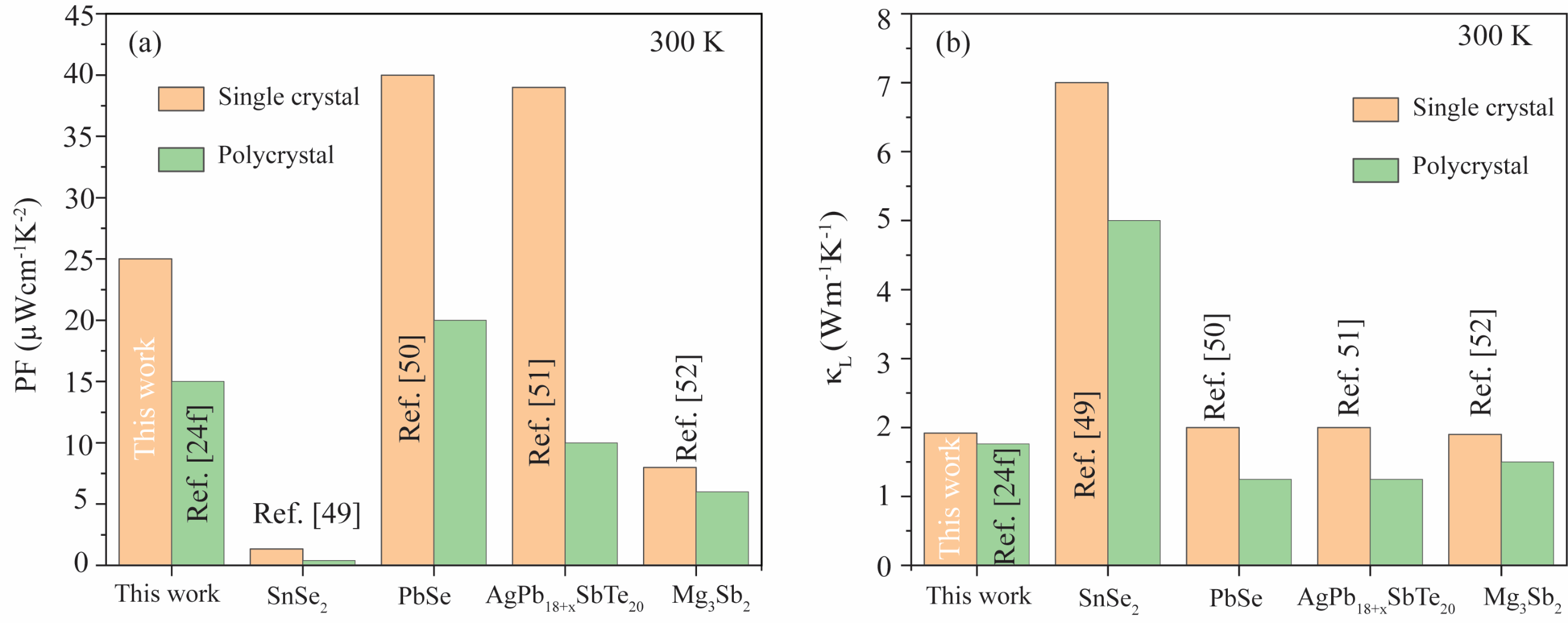


**Figure S25.** A comparison of several crystal ingots obtained by temperature gradient method (a) power factor (b) lattice thermal conductivity.


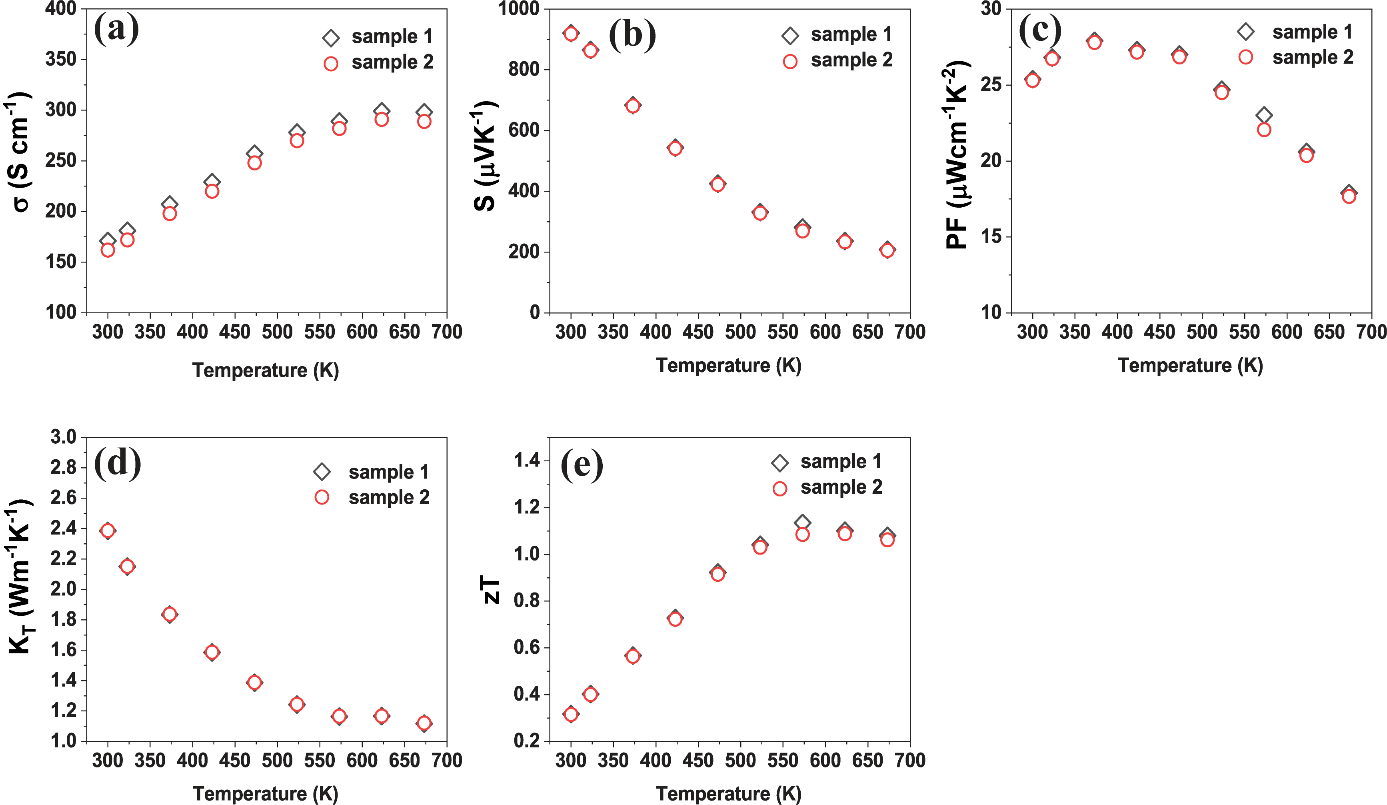


**Figure S26.** Reproducibility of thermoelectric properties in the parallel (⫽) direction for Te_1-x_(Sb)_x_ (a) electrical conductivity (b) Seebeck coefficient (c) power factor(d) total thermal conductivity (e) lattice thermal conductivity (f) figure of merit, zT.

**Supplementary note on synthesis of polycrystalline Te by spark plasma sintering**

For comparison, polycrystalline ingots were synthesized by spark plasma sintering (SPS). The Te shots were sealed in a quartz tube and placed in a horizontal furnace. The samples are heated to 510$℃$ and allowed to dwell for 12 h. Finally, it is furnace cooled to room temperature. The obtained ingot is pulverized in an agate mortar and sieved. The powder is then plugged into a graphite mold of diameter 12.7 mm and sintered (SPS-211Lx, Dr. Sinter Lab) in a vacuumed chamber at 673 K for 10 min with a uniaxial pressure of 50 MPa. The electrical and Seebeck coefficients samples with dimension of $3\times3\times10$ mm and thermal conductivity samples with dimension of $8\times8\times2$ were obtained in parallel (⫽) and perpendicular directions (┴).


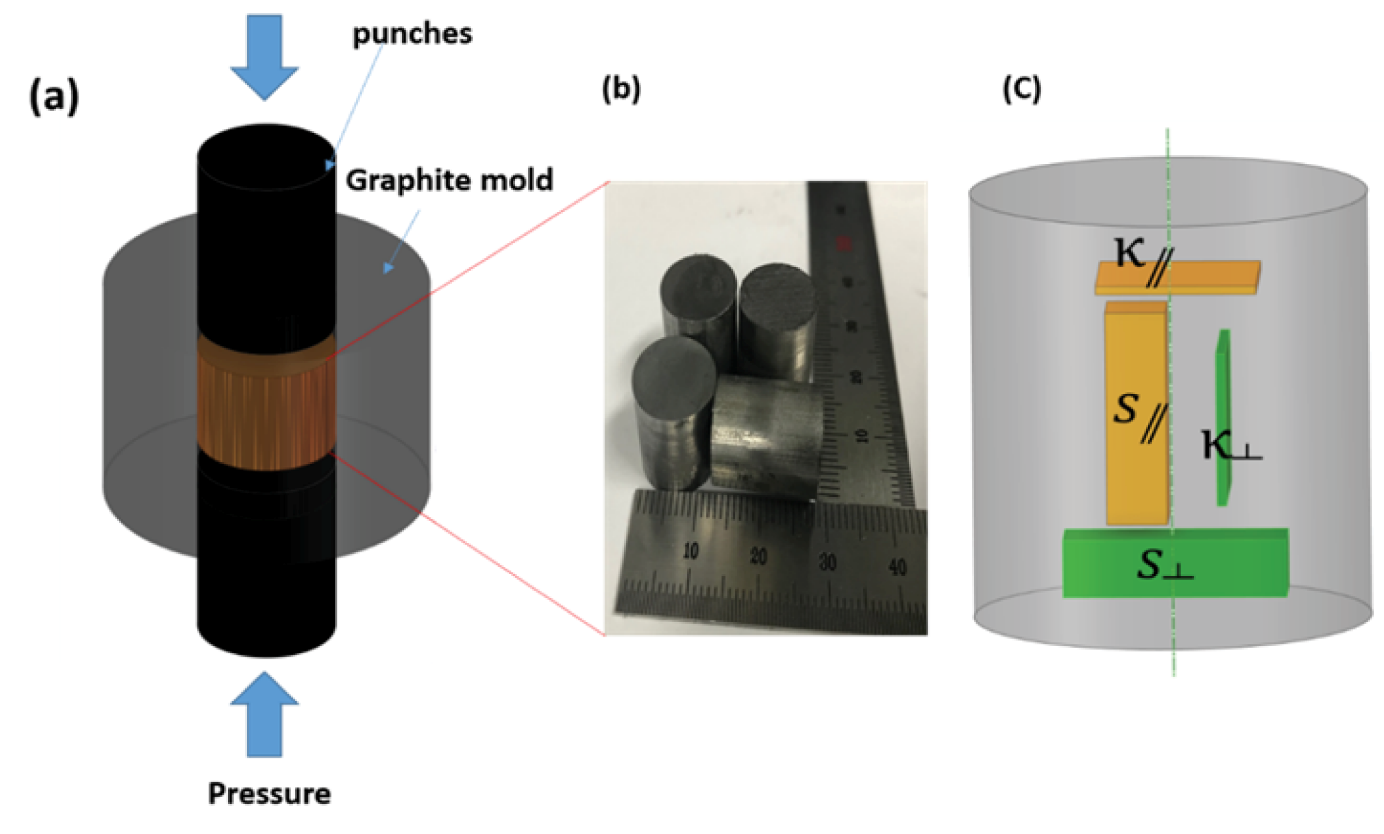


**Figure S27**. (a) Spark plasma sintering technique (b) SPS of pure Te (c) Anisotropic measurement of thermoelectric properties.


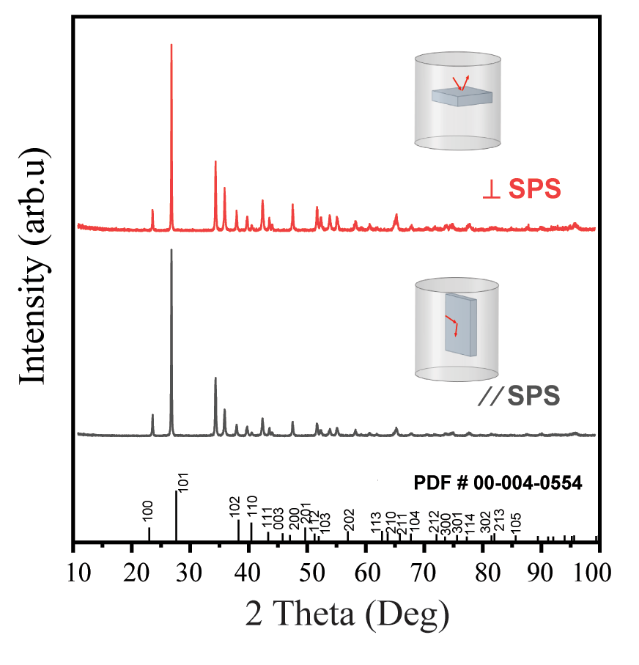


**Figure S28**. XRD profile of Spark plasma Sintered samples in the parallel (⫽) and perpendicular direction (┴)

**Calculations**

**Calculation of orientation by Lotgering factor**

$LF=\frac{P-P_{0}}{1-P_{0}}$ *(1)*

$P_{0}=\frac{I_{0}(00l)}{Ʃ I(hKl)}$ *(2)*

$P=\frac{I_{0}(00l)}{Ʃ I(hKl)}$ *(3)*

$P$Where *I* and $I_{0}$represent the peak intensity measured for sample and reference data

**Calculations by Single Parabolic Model (SPB)**

The carrier effective mass of Te can be calculated based on the measured Seebeck coefficient and carrier concentration assuming acoustic phonon scattering. The equations are expressed by simplified single parabolic band model.

$s=S_{o}\times In\left( 1.075+\frac{e^{2}}{n_{r}} \right)$ *(4)*

$n_{r}=\frac{2}{\sqrt{\pi}}F_{1/2}\left( \eta\right)$ *(5)*

$n=n_{m0}\left( \frac{{m^{*}T}/K}{m_{e} 300} \right)^{1.5}\times n_{r}$ *(6)*

$\left( \frac{{m^{*}T}/K}{m_{e} 300} \right)^{1.5}=\frac{n}{n_{m,0}}\left[ exp\left( \frac{S}{S_{0}}-2 \right)-0.1455 \right]$ *(7)*

$\mu_{o}=\mu\times\left[ 1+\left( \frac{{m^{*}T}/K}{m_{e} 300} \right)^{1.5}\frac{n/{cm}^{-3}}{2n_{m,0}} \right]^{\frac{1}{3}}$ *(8)*

Where $S_{o}=86.1733 \mu V/K$ and $n_{m0}=2.5094\times{10}^{19}{cm}^{-3}$

**Calculation of electronic quality factor B_E_:**

$B_{E}=S^{2}\sigma/\left[ \frac{S_{r}^{2}exp(2-S_{r})}{1+exp\left[ -5\left( S_{r}-1 \right) \right]}+\frac{S_{r}\frac{\pi^{2}}{3}}{1+exp\left[ 5\left( S_{r}-1 \right) \right]} \right]$ *(9)*

**Goldsmid Sharp formula**:

$E_{g}=2eS_{max}T$ *(10)*

Where $S_{max}$ is the maximum Seebeck coefficient, T is max temperature.

**Thermoelectric conversion Efficiency (η) can be calculated from the formula**[83]**:**

$\eta=\frac{T_{h}-T_{c}}{T_{h}}\frac{\sqrt{1+zT_{ave}}-1}{\sqrt{1+zT_{ave}}+{T_{c}}/{T_{h}}}$ *(11)*

Where $T_{h}$ and $T_{c}$ are the hot and cold side temperature, respectively.

The average Z is obtained by

$$Z_{int}=\left( 1/{\Delta T} \right)\int_{T_{c}}^{T_{t}} Z\left( T \right)dT$$

$Z_{Tavg}=Z(T_{avg})$ *(12)*

**Minimum lattice thermal conductivity by Cahill**[80]

$\kappa_{min}=\frac{K_{B}}{2.48}p^{\frac{2}{3}}(\nu_{l}+2\nu_{t})$ *(13)*

Where $\nu_{l}$ and $\nu_{t}$ is the longitudinal and transverse sound velocity.

**Calculation of Lattice thermal conductivity by Debye Callaway Model**

Theoretical calculations of lattice thermal conductivity based on the modified Callaway model is given by:

$K_{l}=\frac{K_{B}}{2\pi^{2}v}\left( \frac{K_{B}T}{\hbar} \right)^{3}\int_{0}^{\frac{\theta_{D}}{T}} \tau_{tot}(x)\frac{x^{4}e^{x}}{(e^{x}-1)}dx$ *(14)*

Where $K_{B}$ is Boltzmann constant,$v$ is an average speed of sound, ℏ is the reduced Planck constant,$\theta_{D}$ is Debye temperature, $\tau_{tot}$ are total relaxation time and x $=\frac{\hbar\omega}{K_{B}T}.$Following Matthiessen’s rule the total relaxation time ($\tau_{tot}$) is a summation of contributions from various Umklapp ($\tau_{U}^{-1}$) and normal$(\tau_{N}^{-1}$) process. In this work, we also considered mainly scattering by point defects $\tau_{PD}^{-1}$ due to solid solution, dislocations core $\tau_{DC}^{-1}$ , and dislocation strain $\tau_{DS}^{-1}$ and precipitate scattering $\tau_{PS}^{-1}$

$\tau_{tot}^{-1}=\tau_{U}^{-1}$+$\tau_{N}^{-1}$+$\tau_{PD}^{-1}+\tau_{DC}^{-1}+\tau_{DS}^{-1}+\tau_{PS}^{-1}$ *(15)*

$\tau_{PS}^{-1}=v_{m}N_{Ps}\left[ \left( 2\pi R_{p} \right)^{-1}+\left( \pi R^{2}\frac{4}{9}\left( \frac{\Delta D}{D} \right)^{2}\left( \frac{\omega R_{p}}{v} \right)^{4} \right) \right]$ *(16)*

$V_{m}=\left( \frac{1}{3}\left( \frac{1}{v_{l}^{3}}+\frac{2}{v_{t}^{3}} \right) \right)^{\frac{1}{3}}$ *(17)*

**Table S3**. Debye -Callaway $\kappa_{l}$ based on phonon scattering mechanism of Umklapp processes (U), normal processes (N), point defects (PD). Dislocations core (DC), and dislocation strain $(DS)$ and precipitate scattering (PS).

| Parameters | **Description** | **Values** |
| --- | --- | --- |
| V($Å^{3}$) | Unit cell volume | 101.81 |
| $\beta$ | Prefactor fitted | 0.6 |
| $V_{L}$ | Longitudinal velocity | 3571 m/s |
| $V_{T}$ | Transverse velocity | 1736 m/s |
| $\gamma$ | Gr$\ddot{u}\mathrm{neisen}$ | 2.4 fitted |
| $\overline{V}$ | Average at. volume | 3.43$\times{10}^{-29}m^{3}$ |
| $\delta$ | Average Atomic mass | 2.11$\times{10}^{-25}kg$ |
| $N_{D}$ | Dislocation density | 1.34 $\times{10}^{11} cm^{-2}$ |
| $\theta_{D}$ | Debye Temperature | 147 K |
| $R_{p}$ | Radius of precipitate | 4 nm (fitted) |
| $N_{ps}$ | Density of precipitate | 7$\times{10}^{23}m^{-3}$ |
| $r_{Sb}$ | Atomic radius of Sb | 1.45 Å |
| $r_{Te}$ | Atomic radius of Te | 1.4 Å |
| d | Grain size | 50 µm (fitted) |
| $M_{Te}$ | Molar mass of Te | 127.6 g/mol |
| $V_{m}$ | velocity of sound | 1950m/s |
| b | Burgers vector | 13.5 (fitted) |
| $v_{p}$ | Poisson ratio | 0.23 |
| D | Density of Te | 6.23 |
| M _Sb2Te3_ | Atomic mass of Sb_2_Te_3_ | 2.07$\times{10}^{25}kg$ |
| $\Gamma$ | Mass fluctuation parameter | 0.2 (fitted) |
